# Responsive self-assembly of tectoRNAs with loop–receptor interactions from the tetrahydrofolate (THF) riboswitch

**DOI:** 10.1093/nar/gkz304

**Published:** 2019-05-02

**Authors:** Charles Mitchell, Julio A Polanco, Laura DeWald, Dustin Kress, Luc Jaeger, Wade W Grabow

**Affiliations:** 1 Department of Chemistry and Biochemistry, Seattle Pacific University, Seattle, WA 918119-1997, USA; 2 Department of Chemistry and Biochemistry, Biomolecular Science and Engineering Program, University of California, Santa Barbara, CA 93106-9510, USA

## Abstract

Naturally occurring RNAs are known to exhibit a high degree of modularity, whereby specific structural modules (or motifs) can be mixed and matched to create new molecular architectures. The modular nature of RNA also affords researchers the ability to characterize individual structural elements in controlled synthetic contexts in order to gain new and critical insights into their particular structural features and overall performance. Here, we characterized the binding affinity of a unique loop–receptor interaction found in the tetrahydrofolate (THF) riboswitch using rationally designed self-assembling tectoRNAs. Our work suggests that the THF loop–receptor interaction has been fine-tuned for its particular role as a riboswitch component. We also demonstrate that the thermodynamic stability of this interaction can be modulated by the presence of folinic acid, which induces a local structural change at the level of the loop–receptor. This corroborates the existence of a THF binding site within this tertiary module and paves the way for its potential use as a THF responsive module for RNA nanotechnology and synthetic biology.

## INTRODUCTION

Naturally occurring ribonucleic acid (RNA) molecules like the ribosome utilize recurrent structural patterns known as modules or motifs to fold and assemble into specific three-dimensional shapes (e.g. (1–7)). The modular nature by which functional RNAs result from the mixing and matching of structural modules provides a blueprint for the rational design of artificial RNA-based nanostructures (8–12). RNA modules that facilitate long-range RNA-RNA interactions are known to play especially crucial roles in the initiation and spontaneous folding and/or self-assembly and for this reason have and continue to provide exemplary sources of inspiration for building artificial nanostructures (e.g. (8,13–15)). Synthetic RNA nanoparticles built from such structural elements have provided promising tools for synthetic biology, cancer, and genetic diseases (10,12,16–19). The continued discovery and characterization of RNA modules with distinctive geometrical and functional properties hold potential for various additional applications. Like many technological fields in our digital age, RNA nanotechnology has and continues to receive a boost from the use of automated computational programs that mine through the rapidly growing number of genomic databases associated with non-protein coding RNAs (ncRNAs) to search for new structural patterns (20–22). A current and rich example of this over the past decade involves the discovery of riboswitches—ncRNA segments that alter gene expression levels through metabolite binding that induces intramolecular transcriptional regulation (23,24).

Given their dynamic and often unique structural features, riboswitches may provide a new frontier for the discovery of novel structural modules with responsive behavior. For example, the previously characterized tetrahydrofolate (THF) riboswitch regulates the translation of enzymes involved in both prokaryotic and eukaryotic folate metabolism using a unique long-range loop–receptor interaction (25–29). Previous functional data also suggest that the THF riboswitch contains two binding sites for various folate derivatives—a unique feature among all other known riboswitches (27,28). One of the two purported binding sites resides at the interface of the loop–receptor interaction and has been implicated most directly in the riboswitch's function.

The discovery of new RNA modules does not ensure their applicability for nanodesign automatically. New RNA components require experimental validation for being incorporated into larger contexts. The rational design of artificial RNA nanostructures built on programmable structural modules can often provide means for the characterization and/or validation of these RNA modules. In this regard, previous works have demonstrated the usefulness of building artificial self-assembling RNA molecules, called tectoRNAs, for assessing the thermodynamic stability, dynamics, self-assembly and folding properties of small RNA structural modules, including various RNA junctions (2,30–32), internal loop motifs (33–35), and various non-covalent long-range interactions such as loop–loop (8,15,36,37) and loop–receptor (8,10,13,33,38–40) tertiary interactions. The functional integration of these RNA modules into minimally designed tectoRNAs provides a straightforward system to assess their ability to direct molecular assembly processes, and answer other questions regarding their specific physical and biological properties outside their larger natural structural/functional contexts (e.g. (2,30,32,37)). Some tectoRNAs have seen considerable use in the characterization of GNRA tetraloops and their corresponding receptors (13,33,37–44). This strategy has provided the basis to select for GNRA and non-GNRA loop–receptors (38,45), to enhance various ribozymes (46,47) and more recently, to retro-synthesize ancestral form of GNRA-receptor interactions from the ribosome (40). Using the long-range loop–receptor interaction originally identified in the THF riboswitch as a centerpiece in the design of self-assembling tectoRNAs, we report the characterization of an entirely different class of loop–receptor module and provide evidence for its role in binding reduced folates. To our knowledge, this work represents the first extensive thermodynamic characterization of tectoRNAs whose loop–receptor interactions are responsive to the binding of a small molecule.

## MATERIALS AND METHODS

### TectoRNA rational design

**Figure 1.**
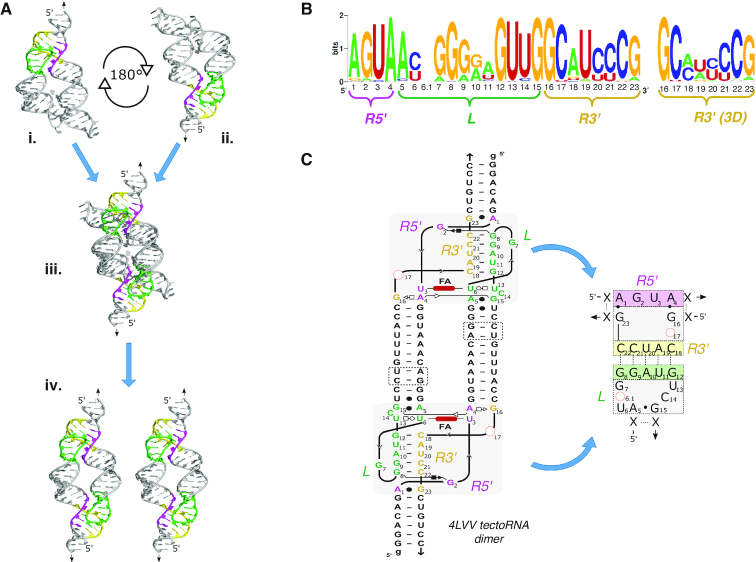
TectoRNA design. (**A**) The crystal structures of two previously reported THF riboswitches (4LVV and 3SUX) were aligned to form THF tectoRNA dimers. The construction of the 4LVV_9 tectoRNA dimer is shown here. The 4LVV crystal structure was inverted, rotated 180°, and then aligned over the original structure. The pink represents the 5′ portion of the receptor. The green represents the nucleotides associated with the loop. The yellow represents the nucleotides found on the 3′ portion of the receptor (see also Supplementary Figure S2). (**B**) The sequence signature of the loop–receptor interaction for the consensus sequence of known THF riboswitches. http://weblogo.berkeley.edu/. (**C**) Full 2D model of the 4LVV tectoRNA and shorthand sequence signature of the 4LVV. Alignment of helices for 4LVV and 3SUX produced an adjoining helix of nine and eleven base pairs between its loop and receptor sequence respectively (see Supplementary Figure S2). The length of tectoRNAs can be altered by removing up to two base pairs on interior helical stem (dashed boxes).

TectoRNAs based on the THF loop–receptor interaction were designed from two previously reported THF riboswitch crystal structures (pdb_IDs: 4LVV & 3SUX) (26,27) using the Swiss-PDBViewer program (48). In each case, the crystal structures of replica images were manipulated to construct tectoRNAs containing a loop and receptor joined by a single helix (Figure 1A). The crystal structures afforded the ability to determine the appropriate length of the helical strut required to facilitate proper loop–receptor orientation and tectoRNA assembly. The sequences associated with the stem were conserved among all constructs and contained the start sequence GGGA required for T7 RNA polymerase. Each sequence was evaluated using Mfold (49), a computational RNA folding program, in order to predict tectoRNA secondary structure and minimize potential unintended intramolecular folding outcomes.

### Nucleic acid synthesis, purification, and labeling

TectoRNAs were transcribed from double stranded DNA (dsDNA) templates using *in vitro* run-off transcription by T7 RNA polymerase as previously described (33) (see also Supplementary data). Individual dsDNA templates were amplified with *Taq* DNA polymerase (Thermo Fisher Scientific) via PCR from synthetically made DNA template and primers (Integrated DNA Technology) according to the manufacturer's protocol. Transcripts were purified through denaturing polyacrylamide gel electrophoresis (PAGE) [8–10% (19:1) polyacrylamide, 8 M urea, 1× TB buffer (89 mM Tris Borate, pH 8.2)]. The resulting gel fragments containing RNA were placed in Crush and Soak buffer (200 mM NaCl, 10 mM Tris pH 7.5, 1 mM filtrated Na_2_EDTA, pH 8), shook overnight at 5°C, and isolated the next day using ethanol precipitation. A portion of each purified transcript was labeled at its 3′ end with [^32^P]pCp as previously described (33) or with the pCp-Cy5 fluorophore (Jena Bioscience). In the latter case, a mixture of 100 pmol of tectoRNA, 0.50 mM pCp-Cy5, and 10 units of T4 RNA Ligase 1 (New England Biotechnology) was incubated at 10°C overnight, heated at 95°C for 3 min, and purified using Invitrogen NucAway™ Spin Columns (Thermo Fisher Scientific). Concentrations of labeled and unlabeled RNA were taken using a Nanodrop 2000 spectrophotometer (Thermo Fisher Scientific). RNA was stored at –20°C prior to use.

### TectoRNA self-assembly and characterization by native polyacrylamide gels

TectoRNA assembly was monitored via gel-shift assays using native PAGE as previously described (2). The equilibrium constant of dissociation (*K*_d_) of each construct was determined by measuring the degree of dimerization as a function of RNA concentration. Each lane contained a fixed amount of labeled RNA (concentrations ranging from 5 to 25 nM) to ensure uniform fluorescent intensity during imaging and increasing concentrations of unlabeled RNA (total concentrations ranging from 5 to 20 000 nM) to impose dimerization. Prior to gel loading, samples were denatured for 2 min at 95°C, snap-cooled for 3 min on ice and incubated in association buffer (89 mM Tris-borate pH 8.2, 50 mM KCl and 2 mM Mg(OAc)_2_) at 30°C for 20 min. Following incubation, the samples (10 μl each) were mixed with 1–2 μl of blue gel loading buffer (1× association buffer, 55% glycerol, 0.1% bromophenol blue) and ran through 7% (29:1) nondenaturing PAGE gels (16.5 cm × 26 cm) at 5 W for ∼3 h in presence of 89 mM Tris–borate pH 8.2 and 2 mM Mg(OAc)_2_, at 7°C. CY5 labeled RNA molecules were visualized using a FluorChem Q fluorescent imaging machine (Protein Simple) and resulting monomer and dimer bands were analyzed using AlphaView SA software. Select samples were also ran in the same buffer conditions as above in the presence of 200 μM folinic acid (FA). Folinic acid was added to each buffer and acrylamide solution at the compensatory expense of dH_2_O, and the results were compared side-by-side with samples lacking folinic acid. *K*_d_ values were experimentally determined by fitting the resulting gel shift assay data to the non-linear equation fD = ((4β*M*_o_ + *K*_d_) – (8*M*_o_β*K*_d_ + *K*_d_^2^)^0.5^)/4*M*_o_, where *M*_o_ is the initial concentration of monomer tectoRNA, fD is the fraction of tectoRNA homo-dimerization, and β is the maximum fraction of tectoRNA that homo-dimerized (50). The change in free energy for each construct was determined using the equation Δ*G* = –*RT* ln *K*_d_, where *R* is the gas constant (1.987 cal·K^−1^mol^−1^) and *T* is the temperature (280 K). Free energy comparisons between tectoRNAs were made using the equation ΔΔ*G* = Δ*G_n_* – Δ*G*_o_, where Δ*G*_o_ represents the free energy of the 4LVV tectoRNA and Δ*G*_*n*_ represents the free energy of a particular experimental construct. The 4LVV tectoRNA was chosen as the reference because of the previously reported crystal structure.

### Lead cleavage probing

TectoRNA assemblies were probed using a lead acetate cleavage assay as described previously (33,38,51). For each assembly, unlabeled tectoRNA at various concentrations ranging from 5 nM to 20 μM was mixed with ∼1 nM of 3′-[^32^P]pCp labeled tectoRNA to monitor assembly and cleavage. For the cleavage assays, tectoRNA assemblies were first denatured at 95°C for 1 min and immediately cooled on ice for 2 min. Using a thermocycler, each tectoRNA sample was incubated at 20°C for 5 min before the addition of association buffer containing 25 mM HEPES pH 7.5, 50 mM KOAc, and either 0.05 mM, 0.5 mM or 2 mM Mg(OAc)_2_ in the presence or absence of 200 μM folinic acid (FA). Following a 30-min incubation period at 20°C, 1 μl of 10 mg/ml tRNA was added prior to addition of 10 mM (final) Pb(OAc)_2_. After 5 min at 20°C, the reaction was quenched with 10 μl of 100 mM EDTA solution. Each sample was ethanol precipitated, washed, and resuspended in colorless loading buffer (10 M urea, 1.5 mM EDTA) prior to loading onto a 10% (19:1) denaturing PAGE gel. Labeled RNA molecules were visualized using a phosphor-screen and a Typhoon Biomolecular Imager (Amersham). Lane profiles and densitometry analysis were analyzed using ImageJ 2.0 software.

## RESULTS AND DISCUSSION

### From crystal structures to tectoRNAs

Previously reported crystal structures of the THF riboswitch (pdb_IDs: 3SUX and 4LVV) reveal that the THF riboswitch forms a long-range Watson–Crick (WC) base pairing interaction known as a pseudoknot (PK) comprised of a terminal loop docked to a complementary receptor (26,27) (Figure 1A and Supplementary Figure S1A). In the full-length riboswitch, the loop and receptor are located on two parallel helices joined by a three-way junction (3WJ) that facilitates a kink turn in the middle of the structure which enables the loop to fold back onto itself and align with the receptor (Figure 1A and Supplementary Figure S1). In the case of the 4LVV crystal structure, the THF riboswitch has two ligand binding sites, one located at the 3WJ sequence and the other within the loop–receptor domain (27). By contrast, the 3WJ ligand binding site is the only one observed in the 3SUX crystal structure (26). It is important to note that the 3SUX crystal structure consists of a dimer of two riboswitches involved in a domain swap occurring near the loop–receptor region. Although this structural rearrangement does not seem to influence the overall orientation of the loop–receptor interaction, it might affect ligand recognition.

In the full-length riboswitch, both the geometry of the 3WJ and the length of the two parallel helices dictate the orientation of the loop–receptor interaction. When the 3WJ is removed (as in the case of the tectoRNA), the orientation of the loop–receptor interaction depends directly on the length of the helix separating the two submotifs from one another (Figure 1). The resulting tectoRNA models suggest that the ideal length separating the loop from the receptor is nine base pairs (bps) for the 4LVV and eleven bps for 3SUX (Figure 1C and Supplementary Figure S2). We theorized that the stem length discrepancy between the two models might result from two primary factors: the inherent flexibility of RNA helices and/or the presence (or absence) of the folate binding pocket. Considering that the pattern of tertiary contacts in the loop–receptor interactions are slightly different between the 4LVV and 3SUX structures (Supplementary Figure S1B), we reasoned that the presence of a folate binding-site in 4LVV could influence the orientation of the loop–receptor interaction and in turn affect the stem length required to provide optimal tectoRNA assembly.

There are at least 57 known unique THF riboswitch sequences in nature but many of these possess conserved sequences within their respective loop–receptor domains (25). When grouped by loop–receptor identity, we found 29 unique loop–receptor sequences variations among the similar sequence signature (Supplementary Table S1, Figure 1B). We noticed that based on the two crystallographic structures, the 3′ portion of the receptor can be realigned to reflect the constraints on the long-range base pairing more accurately (Figure 1B). The nomenclature of the naturally occurring sequences are based on the accession numbers used in Ames *et al.* (25) where the last three digits of each code were used as short-hand identifiers of each unique loop–receptor sequence signature (e.g. 980, 593, 158, etc.). By default, all the constructs derived from natural THF riboswitches refer to constructs with the 11-bp stem connector (e.g. the construct ‘980’ refers to the tectoRNA with the loop–receptor 980 and an 11-bp stem connector). Constructs with 9- and 10-bp connectors are indicated by the base pair designation after the identification number respectively (i.e. 980_9 refers to the 980 sequence with a 9-bp stem connector). Finally, specific artificial variants derived from a particular natural loop–receptor THF motif are indicated as follows: 980.1, 980.2 or 4LVV.1, 4LVV.2, etc. The complete list of sequences, their groupings, and names are detailed in the Supplementary Data information (Supplementary Tables S1 and S4).

**Figure 2.**
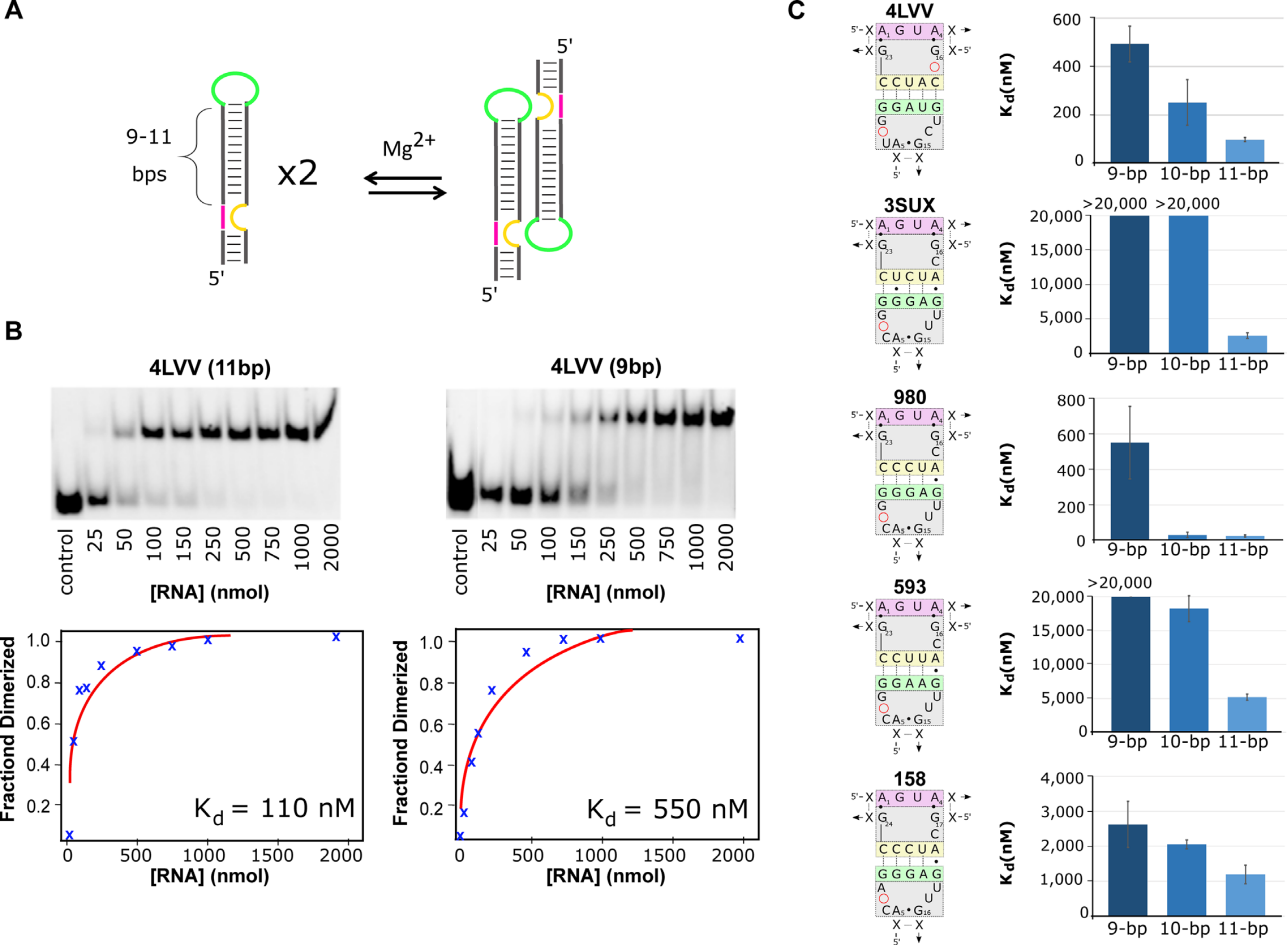
TectoRNA assembly. (**A**) Schematic of tectoRNA assembly based on dimerization of tectoRNA monomers. (**B**) The tectoRNAs were incubated at increasing monomer concentrations until equilibrium between monomer and dimer populations was established in presence of 2 mM Mg(OAc)_2_ at 7°C. Monomer and dimer populations of tectoRNAs were visualized by gel shift assays. Subsequent analysis provided a graphical dissociation constant at equilibrium (*K*_d_). Lane labelled ‘control’ involves a size marker corresponding to an inactive THF tectoRNA (at 50 nM). (**C**) Sequence signatures of the loop–receptor interaction for the five tectoRNAs tested with varying stem lengths and the corresponding *K*_d_ for each stem length. All data represent the average and standard deviation from a minimum of three independent experiments.

The self-assembly properties of tectoRNAs bearing the three most conserved loop–receptors interactions (980, 593, 158) along with those from the two reported crystal structures (3SUX and 4LVV) were tested by native poly-acrylamide gel shift assays at varying RNA concentrations (Figure 2). As the tectoRNA concentration increases, tectoRNA dimers form and migrate more slowly than monomers, allowing equilibrium constants of dissociation (*K*_d_) to be determined (Figure 2 and Supplementary Table S2). TectoRNAs with the lowest *K*_d_s exhibit the strongest loop–receptor interactions. We initially tested constructs in presence of 15 mM Mg(OAc)_2_ (which was the same Mg^2+^ concentration used previously for GNRA/receptor tectoRNA systems (2,37,38)) but the 4LVV and 980 tectoRNAs were dimerizing below our detection limits—preventing reliable K_d_ measurements. By lowering the magnesium ion concentration to 2 mM in the association buffer (89 mM Tris-borate, pH 8.2, 50 mM KCl and 2 mM Mg(OAc)_2_), the self-assembly of tectoRNAs with the strongest interactions could be differentiated at RNA concentrations >1 nM. Subsequently, *K*_d_ measurements were typically performed at 2 mM magnesium concentration and at 7°C.

In order to find which stem length provided optimal conditions for dimer formation, the initial five tectoRNAs (4LVV, 3SUX, 980, 593 and 158) were each tested with stem connectors of 9, 10 and 11 bp (Figure 2). Overall, the tectoRNAs possessing the 11-bp stem self-assembled more efficiently than their 9- and 10-bp counterparts (Figure 2C and Supplementary Table S2). *K*_d_s for 10-bp tectoRNAs were somewhat between those observed for the 9- and 11-bp tectoRNAs. This suggests that the longer stem provides optimal geometry between the loop–receptor interactions. Nevertheless, the extent of the improvement in binding affinity varied significantly depending on the loop–receptor context. For instance, from 9- to 11-bp stem connectors, the *K*_d_s of 158 related tectoRNAs improved by about a factor of two whereas those of 4LVV related tectoRNAs improved by about a factor of five. Moreover, connectors of 10- and 11-bps led to almost identical *K*_d_s in the context of the loop–receptor 980. The lowest *K*_d_s were obtained with 980 and 4LVV tectoRNAs, which dimerized with K_d_s of 26 (±6) and 98 (±8) nM, respectively (Figure 2C, Supplementary Table S2). The loop–receptor sequences of 980 and 4LVV differ at eight nucleotide positions. Interestingly, in the 11-bp connector context, 158 and 3SUX (which both differ from 980 by only one point mutation in the loop or receptor) assembled with *K*_d_s ∼ 50–100 times higher than 980. Moreover, 593 (which differs from 980 by having G10:C20 bp changed into A10:U20 bp in the long-range pairing) assembled with a *K*_d_ 200 times higher than 980 (Figure 2C, Supplementary Table S2). These data demonstrate that efficient tectoRNA self-assembly in absence of ligand is influenced by connector stems of 11-bp but is also highly dependent on the loop–receptor sequence.

Given that our initial model for the 4LVV tectoRNA dimer indicated an optimal stem length of 9-bp rather than 11-bp, our results indicate that in the absence of a folate derivative the loop–receptor conformation is likely sub-optimally orientated. Our data suggest that riboswitches may not be optimized for producing the strongest loop–receptor interactions (at least in the absence of folate derivatives). This makes sense given the nature of riboswitches, which consist of dynamic structures that are required to oscillate between alternative structures to facilitate metabolite sensing. Selection pressures would not be expected to create the strongest binding conditions as this would result in riboswitch structures that would be trapped in one conformation and thus prevent its structural rearrangement into another functional form. Additionally, these data also suggest that minimal changes in the THF loop–receptor sequence can provide dramatic modulation with respect to their ability to self-assemble.

### Characterization of the THF Loop-Receptor sequence signature

**Figure 3.**
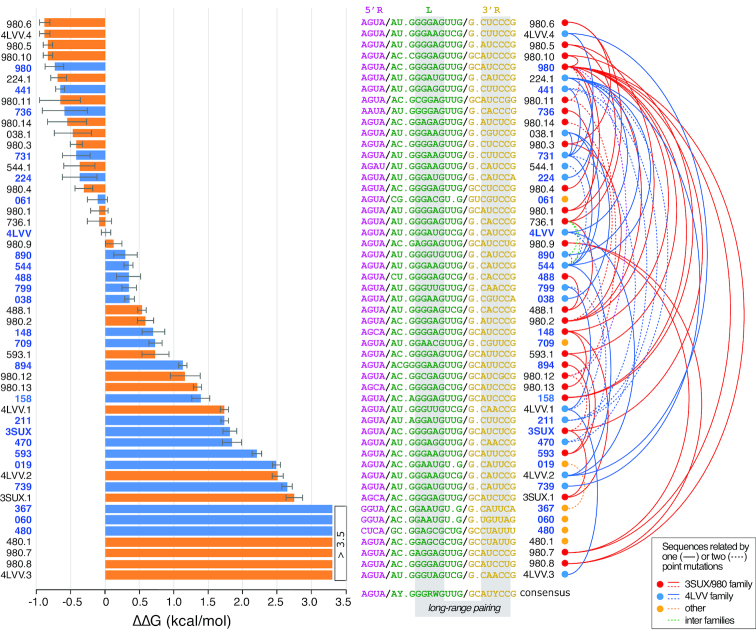
Probing the thermodynamics of the THF loop–receptor interaction. Variant constructs based on dimer formation from tectoRNA assembly were tested against 4LVV. ΔΔG were determined as indicated in the Materials and Methods in presence of 2 mM Mg(OAc)_2_ at 7°C. TectoRNAs with naturally occurring sequences are shown in blue and artificial variants are shown in orange on the bar graph. Corresponding sequences are shown next to the alphanumeric identifiers of each unique tectoRNA sequence. For the consensus sequence, each position is at least 75 conserved (see also Supplementary Table S1 for more details). R = A or G; Y = U or C; W = A or U.

Based on the above findings, we decided to characterize the binding affinities of naturally occurring loop–receptor interactions as well as targeted artificial sequences within the context of the 11-bp tectoRNA design (Figure 3, Supplementary Tables S1 and S3). Out of the 51 tectoRNAs tested, 46 are related to another sequence by two point mutations or less and can be approximately grouped into two sequence families (Figure 3 and Supplementary Figure S3): the 3SUX/980 and 4LVV families comprising 26 and 18 of the total unique sequences, respectively. By comparing the binding affinities of these sequences, we aimed at investigating the sequence signature of the THF loop–receptor to pinpoint critical nucleotides required for tectoRNA assembly (see Figure 1B). Free energies of homodimer formation (Δ*G*) were derived from equilibrium constants of dissociation determined by gel-shift assays at 2 mM Mg(OAc)_2_ and 7°C. The relative strength of each loop–receptor interaction, expressed as a ΔΔ*G*, was estimated using the Δ*G* of 4LVV as the reference (see Figure 3, Materials and Methods and Supplementary Table S4). Using this strategy, we sought to identify nucleotide positions that were most critical to the THF loop–receptor interaction's performance.

Characterization of the individual THF loop–receptor interactions and their respective ΔΔG’s reveal a number of important insights about the motif's comprehensive sequence signature (Figures 1B, 3, and Supplementary Figure S3A). As expected, the tertiary interaction is directly dependent upon the presence of classic WC bps (G:C and A:U bps) which occur between the loop positions (L) 8–12 and positions 18–22 of the 3′ strand of the receptor (R3′) (Figure 1C). The interaction, however allows for a significant number of G:U bps and mismatches (e.g. A:A and A:G bps) with some positions being more tolerant to these non-canonical bps than others. For instance, a G:U bp at position 8:22 (e.g. 980.8) is more detrimental to the interaction's behavior than within positions 9:21 (e.g. 3SUX, 739) and 10:20 (593.1 and 890) (Figure 3 and Supplementary Figure S3). Overall, non-classic WC combinations within bps involving positions 8–10 of the loop and positions 20–22 of R3′ (see Figure 1BC) were found to be more unfavorable to self-assembly than those within bps involving positions 11–12 of the loop and positions 18–19 of R3′ (Figure 3 and Supplementary Figure S3A). For example, a G:U bp within positions 8–9:22–21 (e.g. 3SUX, 739 and 980.8) more severely destabilizes the loop–receptor interaction than a G:U bp or G:A and A:A mismatches within positions 11–12:19–18 (e.g. 980, 544.1, 038.1, 441 and 980.5). This asymmetry is consistent with the x-ray structure of 4LVV, which reveals strong tilting between base pairs 10:20 and 11:19 and base pairs 11:19 and 12:18 to better accommodate non-canonical base pairs in this part of the long-range base pairing (27). In conjunction with their localization within the long-range base pairing, tolerance for G:U bps or mismatches also seems to depend on the total number of G:C bps involved in the loop–receptor base pairing. In general, loop–receptors with three or more G:C bps display greater tolerance for non-classic WC combinations within positions 11–12:19–18. For example, 488.1, which comprises four G:C bps in its loop–receptor base-pairing, is nominally affected by it's A:A mismatch in position 11:19, whereas 4LVV.2, which comprises the same mismatch but only three G:C bps, shows a much lower binding affinity (Figure 3 and Supplementary Figure S3A, Table S4). Comparison of binding affinities of 4LVV to 4LVV.1, or of 4LVV.2 to 4LVV.3 also demonstrates that the loop–receptor base pairing prefers purines over pyrimidines in the loop strand. For example, swapping the A10:U20 bp of 4LVV into U10:A20 (4LVV.1) or the A10:U20 bp of 4LVV.2 into U10:A20 (4LVV.3) dramatically impairs self-assembly (Figure 3 and Supplementary Figure S3A). However, the effect on self-assembly of a bp swap is dependent on its location within the loop–receptor base pairing. For example, swapping the G8:C22 bp of 980 into a C8:G22 bp (980.11) is much less detrimental to self-assembly than swapping the G9:C21 bp of 980 into a C9:G21 bp (890.12) (Supplementary Figure S3A).

The thermodynamic stability of a particular long-range pairing can be significantly affected by its surrounding structural context, notably nucleotides at positions 6, 14 and 17 (Supplementary Figure S3A, B). The context shared by most natural receptors is the U6/U14/del17 context (e.g. 441, 731, 890, 799, 544, 709, 470, 739, 224, etc.) followed by the C6/U14/C17 context (e.g. 3SUX, 980 and 593) and the U6/C14/del17 context (e.g. 4LVV and 488) (Supplementary Figure S3A). Whereas identical long-range pairings in the C6/U14/C17 and U6/U14/del17 contexts displayed similar thermodynamic stability (e.g. 980 and 980.5, 980.4 and 980.6), marked differences were observed for long-range pairings in the U6/C14/del17 context (Supplementary Figure S3A). For instance, identical pairings tend to be thermodynamically less stable in the U6/C14/del17 context than in the U6/U14/del17 context (e.g. 4LVV.1 and 799, 488.1 and 736.1, 4LVV.2 and 544) despite the fact that these two contexts differ only at position 14. This is somewhat unexpected as, in the 4LVV crystallographic structure, C14 is shown bulging out and is not implicated in any tertiary contact with another nucleotide (27). However, in the 3SUX crystallographic structure (26), U14 might form a partial contact with A5, suggesting that the presence of a U might be preferred over a C at position 14. It is possible that in absence of folinic acid, the THF loop–receptor interaction could adopt a conformation closer to the one shown in the 3SUX crystallographic structure. Alternatively, the effect of the surrounding context on assembly might well be indicative of the dynamic and transient involvement of some nucleotides in the formation of the loop–receptor interaction. As seen with variant 061, the absence of nucleotide at position 14 does not seem to affect much self-assembly when this change is associated to A5C and C6G (Supplementary Figure S3C).

**Figure 4.**
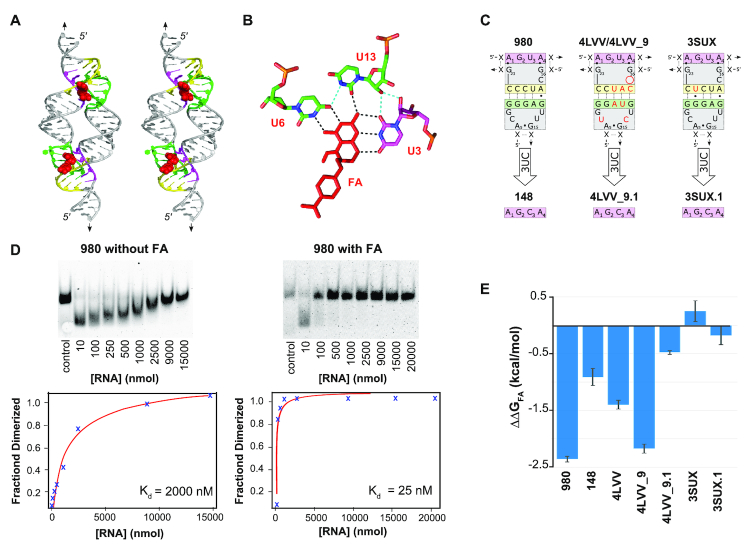
THF TectoRNA assembly is responsive to folinic acid (FA). (**A**) Stereo image of tectoRNA model built from 4LVV_9 shows the binding site of folate (in red) at the loop–receptor interface. (**B**) Three uracil residues are shown to be responsible for interacting with the folate derivatives. Positions U3, U6 and U13 in the loop–receptor correspond to positions U7, U42 and U35, respectively, in the THF riboswitch X-ray structure 4LVV. Hydrogen bonding shown in black is between THF loop–receptor residues (green and magenta) and FA (red). Hydrogen bonding between U3 and U13 is shown in light blue. (**C**) 2D diagram of the various loop–receptor tested in presence (and absence) of FA. Nucleotide positions in red in 4LVV and 3SUX are those that differ from 980. (**D**) Gel shift assays showing the dimerization of the 980 tectoRNA variant and its subsequent K_d_ analysis graphs in absence (top) and presence (bottom) of 200 μM FA. Control lane involves a size marker corresponding to a fully dimerized THF tectoRNA. (**E**) Summary of ΔΔ*G*_FA_ data documenting the effect of FA on binding energy (see also Supplementary Table S3).

Overall, most of the sequence variations at loop–receptor positions other than those involved in the long-range pairing had a relatively mild effect on tectoRNA self-assembly (Figure 3 and Supplementary Figure S3B). The similar thermodynamics for long-range pairings within the C6/U14/C17 and U6/U14/del17 suggest that the variations C6U and C17del synergistically work to restore or reinforce binding. This is supported by the fact that when the changes C6U and C17del occur separately as in the case of 980.1 and 980.2, these changes tend to increase *K*_d_s by 3- to 12-fold with respect of 980 or 980.5 (Supplementary Figure S3B). The change U3C in 3SUX and 980 decreased the strength of the loop receptor interaction by 5- to 13-fold, respectively (see 3SUX versus 3SUX.1 and 980 versus 148, Supplementary Figure S3B). This position is directly involved in the recognition of THF derivatives and makes a ribose zipper contact with U13 (Figure 4B). However, there is no obvious reason why the U3C change would affect self-assembly in absence of folinic acid, unless this position might eventually be prone to adopt alternative conformations that could partially impede formation of the loop–receptor interaction. While the changes G2A and A5C led to 1.5- to 2-fold decrease of *K*_d_ (see 736 versus 736.1 and 488 versus 488.1 Figure 3B), G23A led to very minor increase of *K*_d_s by 2- to 3-fold (see 224.1 versus 224 and 038.1 versus 038 Figure 3B). This indicates that these positions are not critical for assembly unless are also associated with other changes (e.g. G23A/A1G for 019 versus 367, Figure 3C). Finally, variations in the highly conserved guanosine at position 7 produced sequence specific effects on assembly: the G7A mutation in 980 (158) was shown to increase the *K*_d_ by 46-fold whereas the G7C change (980.10) had no effect (Figure 3B). Moreover, tectoRNA 894 has an additional G (G6.1) between position 6 and 7 and self-assembles with a lower *K*_d_ than 593 (Supplementary Figure S3C), suggesting that this region might have some degree of structural plasticity to accommodate extra nucleotides.

In summary, our data emphasize that relatively few point mutations within the sequence signature of the THF loop–receptor interaction can offer an extensive range of *K*_d_s for modulating the self-assembly property of the THF riboswitch. Moreover, the trends identified provide a framework for understanding and predicting the influence that a given point mutation may have on the relative strength of a given interaction.

### Folinic acid and tectoRNA assembly

Whereas the full-length THF riboswitch takes advantage of two structurally distinct binding sites for THF or other derivatives such as folinic acid (FA)(27,28), the THF tectoRNA relies on the synergistic assembly of two identical loop–receptors potentially bearing a FA binding site at each of the two the loop–receptor interfaces (Figure 4A). Therefore, the THF tectoRNA system is particularly suited for exploring the responsive behavior of the THF loop–receptor in presence of FA (Figure 4A and Supplementary Table S3). Because 980 and 4LVV dimerize at 2 mM Mg^2+^ with *K*_d_s close to the detection limit of the gel shift assay, self-assembly experiments on these tectoRNAs and derivatives (148, 4LVV_9 and 4LVV_9.1) were instead performed at 1 mM Mg^2+^ in order to more definitively detect any potential enhancement of FA on tectoRNA assembly. In these conditions, 980, 4LVV and 4LVV_9 assembled in absence of FA with K_d_s of 1944 ± 108, 1072 ± 20 and 3694 ± 432 nM, respectively. The 3SUX tectoRNA and its derivatives did not assemble at 1 mM Mg^2+^ and were therefore tested at 2 mM Mg^2+^ as in the previous cases.

The presence of 200 μM of FA significantly lowered the K_d_ of 4LVV, 4LVV_9 and 980 by a factor of 12- to 70-fold (Supplementary Table S3 and Figure 4D,E). This strongly supports the direct involvement of reduced folate derivatives in the stabilization of the THF loop–receptor interaction. Furthermore, the 4LVV_9 tectoRNA (with a 9-bp connector stem) assembled with a *K*_d_ ∼3-fold higher than 4LVV (with a 11-bp connector stem) in absence of FA, but had a similar *K*_d_ to its 11-bp counterpart in presence of FA (74 ± 6 nM compared to 4LVV’s *K*_d_ of 86 ± 12 nM). As such, 4LVV_9 displayed a much greater response to the presence of FA than 4LVV. These data are consistent with our initial 3D model of the 4LVV tectoRNA that suggests that, in presence of FA, a 9-bp connector stem might be preferable over an 11-bp stem for forming the loop–receptor self-assembling interface (Figure 4A and Supplementary Figure S2).

According to the X-ray structure of the 4LVV loop–receptor interaction, the uracil residue at position 3 (U3) in the R3′ receptor region makes important hydrogen bonding contacts with FA and U6 and U13 (Figure 4B). Previous studies have indicated that the mutation of this uracil into a cytosine (U3C) disrupts FA binding and inhibits THF riboswitch activity (27,28). In the context of tectoRNA assembly, the variants 4LVV_9.1 and 148, which both contain the U3C mutation, showed a greatly reduced responsiveness to FA in comparison to their respective 4LVV_9 and 980 counterparts (Figure 4CE). In the case of 3SUX and its U3C variant (3SUX.1) however, no significant change in dimer formation was observed in the presence or absence of folinic acid (Figure 4E). This finding is consistent with the absence of a FA binding site in the loop–receptor of the 3SUX crystallographic structure (26) but is somewhat surprising as 3SUX differs from 980 at only one nucleotide position within the long-range pairing that is somewhat localized apart from the set of nucleotides known to directly contribute to the formation of the folate binding pocket (Figure 4C). Therefore, the fact that 980 is responsive to FA whereas 3SUX is not, suggests that small sequence variations away from the metabolite-binding site can still dramatically affect the folding, formation, and integrity of the FA binding site. It remains however to be seen whether this remarkable difference in behavior is generalizable to all THF derivatives.

### Monitoring THF tectoRNA responsiveness to folinic acid by lead structural probing

**Figure 5.**
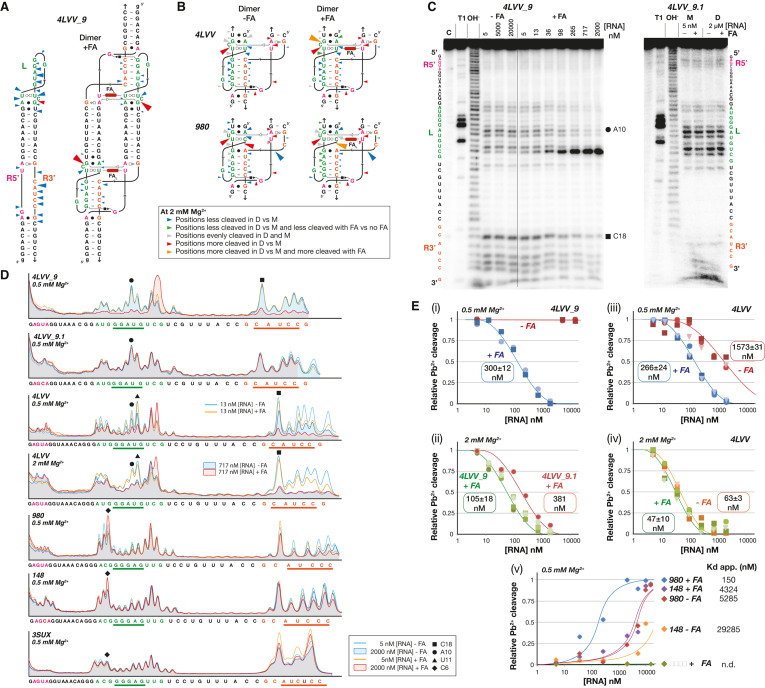
Lead-induced cleavage probing of THF tectoRNAs corroborates that self-assembly is responsive to FA and reveals that binding to FA induces local structural changes. (**A**) Cleavage patterns for 4LVV9 in the monomer state (without FA) and dimer state (with FA) are indicated on tectoRNA 2D diagrams: phosphate positions that are more cleaved in the monomer state than in the dimer state are indicated by blue arrows, those that are more cleaved in the dimer state than in the monomer state are in red. The size of the arrows is roughly proportional to the intensity of cleavage. (**B**) Cleavage patterns for the 4LVV and 980 loop/receptor interactions in presence (right) and absence (left) of FA. Phosphate positions that are less cleaved in the dimer state with FA than without FA are in green, and those that are more cleaved in the dimer state with FA that without FA are in orange. Red and blue arrows (see above); Grey arrows: positions that are evenly cleaved in the monomer and dimer states. (**C**) Typical examples of lead cleavage patterns visualized by denaturing PAGE gels for 4LVV9 and 4LVV9.1 at 0.5 mM Mg(OAc)_2_ (see also Supplementary Figure S4). Cleavage experiments were carried out with 10 mM Pb(II) for 5 min at various concentrations of Mg(OAc)_2_ at 20°C as described in the Materials and Methods section. Lanes labeled C, OH- and T1 correspond to no reaction, alkaline treatment (cleaves at every position) and digestion with RNase T1 (cleaves after G residues), respectively. M and D are for monomer state and dimer state, respectively. (**D**) Lead cleavage profiles for 4LVV9, 4LVV9.1, 4LVV, 980, 148 and 3SUX tested at two different RNA concentrations (5 nM and 2 μM) in absence or presence of 200 μM FA, at 20°C. Profiles were obtained at the indicated Mg(OAc)_2_ concentration (0.5 or 2 mM). Lead cleavage products at several phosphate positions (e.g. C6, A10, U11, C14, C18) are altered upon dimerization and FA addition. Lead cleavage intensity at positions located within the THF long-range interaction (C6 (black diamond), A10 (black circle), U11 (black triangle), C18 (black square)) were quantified and used to estimate *K*_d_ values for tectoRNA self-assembly. (**E**) Plots of the relative fraction of monomer estimated from lead cleavage intensity at positions C6, A10, U11 or C18 in function of tectoRNA concentration in absence or presence of 200 μM FA. Maximum and minimum band intensities at positions A10, U11 or C18 from 4LVV9, 4LVV9.1 and 4LVV were estimated to correspond to the full monomer and full dimer state, respectively. For position C6 of 980, 148 and 3SUX, minimum and maximum band intensities correspond to the full monomer and full dimer, respectively. Solid lines represent theoretical curves for tectoRNA self-assembly at the indicated K_d_ values. K_d_s measured in presence of FA are also indicative of the affinity of FA for the THF loop/receptor interaction. (i) Plot for 4LVV9 in absence and presence of FA at 0.5 mM Mg(OAc)_2_. (ii) Plot for 4LVV9 and 4LVV9.1 in presence of FA at 2 mM Mg(OAc)_2_. (iii, iv) Plot for 4LVV in absence and presence of FA at (iii) 0.5 mM Mg(OAc)_2_ and (iv) 2 mM Mg(OAc)_2_. (v) Plot for 980, 148 and 3SUX in absence and presence of FA at 0.5 mM Mg(OAc)_2_.

Additional structural insights on THF tectoRNAs were gained through lead cleavage experiments performed on 4LVV, 4LVV_9, 4LVV_9.1, 980, 148 and 3SUX at various RNA and magnesium concentrations in the presence and absence of FA at 20°C (see Materials and Methods, Figure 5 and Supplementary Figure S4). Lead generally does not bind or cleave the phosphodiester backbone in regular A-form helical regions of RNA. Lead rather, preferentially cleaves RNA backbone regions that are single stranded, kinked or generally more flexible and accessible to the solvent (33,37,38,51). In conditions preventing self-assembly (i.e. low RNA concentrations (5 nM), low Mg^2+^ concentrations (0.05 mM Mg(OAc)_2_, and/or in the absence of FA), loop and receptor regions were accessible to lead cleavage while the stem regions of the THF tectoRNAs were mostly protected. Our results in these conditions demonstrate that the THF tectoRNAs are in the monomer state: whereby they are folded into their expected secondary structures as predicted by Mfold, with the loop (L) and receptor (R) regions remaining essentially single stranded (Figure 5 and Supplementary Figure S4). By contrast, in conditions that promoted self-assembly (i.e. at high RNA concentrations, high Mg^2+^ and/or presence of FA), significant changes of lead cleavage patterns could be observed in the L and R regions for most of the tectoRNAs investigated.

The most dramatic effects were observed for 4LVV_9 (Figure 5AC). In the absence of FA, lead cleavage patterns for 4LVV_9 remained unchanged at RNA concentrations ranging from 5 nM to 20 μM, indicating that 4LVV_9 essentially remained in its monomer state without FA. By contrast, the L and R3′ regions (both of which are involved in the formation of the long-range base pairing) showed significant protection from lead cleavage in the presence of 200 μM FA (e.g. 3′ phosphate linkages after G9–U11 and C18–C22) (Figure 5A–D), corroborating tectoRNA self-dimerization upon addition of FA. Moreover, the FA binding site within the loop/receptor interaction displayed great responsiveness to FA (Figure 5A–D). Upon increase of RNA concentration and addition of FA, cleavage of the 3′ phosphate linkages after A5, U6 and U13 was slightly reduced, whereas the one after the bulging nucleotide C14 was dramatically enhanced, suggesting that this region undergoes a local conformational change upon FA binding. The variant 4LVV_9.1, which cannot recognize FA as well as 4LVV_9, did not self-assemble with FA at 0.5 mM Mg^2+^ (Figure 5C, D). However, a change of cleavage patterns was observed upon increase of RNA concentration at 2 mM Mg^2+^, indicating that 4LVV_9.1 could nevertheless self-assemble in the presence of FA. These data suggest that the FA binding site of 4LVV_9.1 does not adopt the stable and rigid conformation of its 4LVV9 counterpart upon binding of FA. Using an approach similar to the one for in-line probing (25,52), cleavage intensities at positions located within the THF long-range interaction (e.g. A10 and C18) were quantified and used to estimate apparent K_d_s for tectoRNA self-assembly in absence and presence of FA (Figure 5E(i,ii)). Assuming a two-state model for the THF tectoRNA assembly, plots of the relative fraction of monomer (estimated from lead cleavage intensities at positions A10 and C18) as a function of tectoRNA concentration indicate that, at 20°C in presence of 200 μM FA, 4LVV_9 self-assembles with *K*_d_s of 300 ± 12 nM at 0.5 mM Mg^2+^ and 105 ± 18 mM at 2 mM Mg^2+^ (Figure 5E(i,ii)). In the absence of FA, *K*_d_s could not be determined for 4LVV_9 by this method, as self-assembly likely occurs above 50 μM. Additionally, whereas 4LVV_9.1 assembly cannot be determined at 0.5 mM Mg^2+^, its *K*_d_ is 381 nM in presence of FA at 2 mM Mg^2+^, 20°C.

As expected, lead cleavage patterns at the level of the loop/receptor interaction of 4LVV followed the same trends as those observed for 4LVV_9 (Figure 5B, D and Supplementary Figure S4B), albeit the changes of cleavage intensity upon addition of FA and increasing RNA concentration were not as pronounced as for 4LVV_9. In contrast to 4LVV_9, 4LVV was able to self-assemble in absence of FA (although not as well as in its presence). At 0.5 mM Mg^2+^ and 20°C, the apparent *K*_d_s are 1573 ± 31 nM without FA and 266 ± 24 nM with FA (Figure 5E, (iii)). By increasing the magnesium concentration to 2 mM Mg^2+^, the apparent *K*_d_s are 63±3 nM without FA and 47 ± 10 nM with FA. In good agreement with our previous native gels data, these results corroborate that 4LVV is not as responsive to FA as 4LVV_9. The calculated variation of free energies in the presence and absence of FA (ΔΔG_FA_) for 4LVV_9 and 4LVV measured at 0.5 mM Mg^2+^ and 20°C were within similar range as those measured at 1 mM Mg^2+^ and 7°C by gel shift assays (←2.817 kcal/mol (0.5 mM Mg^2+^, 20°C) versus –2.17 kcal/mol (1 mM Mg^2+^, 7°C) for 4LVV_9; –1.00 kcal/mol (0.5 mM Mg^2+^, 20°C) versus –1.40 kcal/mol (1 mM Mg^2+^, 7°C) for 4LVV). These data also suggest that the 9bp stem construct might be more optimal in performing an induced structural fit upon recognition of FA than its 11 bp counterpart. Additionally, for both 4LVV and 4LVV_9, the marked increase of cleavage intensity at C14 is specific to the presence of FA, suggesting that FA induces specific local conformational change for the nucleotides directly involved in the formation of the FA binding pocket within the THF loop/receptor (Figure 5A, B, D). While C14 is bulging out in the 4LVV atomic structure and is therefore not directly involved in the binding of FA, its ribose-phosphate conformation is critical for structuring the adjacent nucleotides, such as U13, which is involved in the specific recognition of FA (27) (Figure 4B). Because of the FA-dependent, induced fit self-assembly of THF tectoRNAs, K_d_s corresponding to tectoRNA homo-dimerization in presence of FA also correspond to the affinity of FA for the L/R interaction.

Like the 4LVV variants, lead induced cleavage of 980, 148 and 3SUX were consistent with the previous data obtained in presence and absence of FA (Figure 5BDE and supplementary Figure S4). While 3SUX did not assemble in the presence or absence of FA in the conditions tested, 980 (and to a lesser extent 148) displayed subtle but significant changes of cleavage patterns at the level of position C6 upon increase of RNA concentration in the presence and absence of FA (Figure 5D). Indeed, the cleavage pattern indicative of the homodimer state was reached at much lower RNA concentration in the presence of FA, suggesting again that FA induces a local conformational change at the level of the THF loop/receptor interaction. For instance, nucleotide position 6 is directly involved in a contact with FA (Figure 4B). However, intensities of cleavage within the L and 3′R regions directly involved in the long-range base pairing of 980 and derivatives were not as pronounced as those for the 4LVV loop/receptor variants, this being likely due to differences in sequence (51). For 980 and derivatives, lead cleavage experiments performed at 0.5 mM Mg^2+^ were more discriminative for distinguishing structural effects upon addition of FA (Supplementary Figure S4A). *K*_d_s determined by monitoring the change of cleavage intensity at position C6 in 980 and 148 were consistent with those previously determined by gel-shift assays (Figure 5E(v) and Supplementary Table S3). For instance, in the presence of FA, 980 assembled with a *K*_d_ of 150 nM—significantly lower than the one of 148 (4324 nM)—which has a weaker ability to bind FA because of the mutation U3C. Moreover, the calculated variation of free energies in the presence and absence of FA (ΔΔ*G*_FA_) for 980 and 148 measured at 0.5 mM Mg^2+^ and 20°C were within the same range as those measured at 1 mM Mg^2+^ and 7°C by gel shift assays (–1.97 kcal/mol (0.5 mM Mg^2+^, 20°C) versus –2.35 kcal/mol (1 mM Mg^2+^, 7°C) for 980; –1.06 kcal/mol (0.5 mM Mg^2+^, 20°C) versus –0.91 kcal/mol (1 mM Mg^2+^, 7°C) for 148).

## CONCLUSIONS

Our study emphasizes that structural data alone are often insufficient for understanding how a particular structural module will behave in different contexts. Newly discovered structural modules still require experimental characterization, as the rules by which they operate are not always completely predictable—even when their sequences are highly conserved. Our experimental analysis of the loop–receptor interactions from the reported crystal structures of two THF riboswitches shows that these loop–receptor interactions have the ability to self-assemble in the absence of FA—although most constructs show an enhanced ability to assemble in the presence of FA. In its natural, cellular setting, the loop–receptor under investigation operates in *cis* as an integrated part of the THF riboswitch. Thus, its assembly is subject to a variety of structural and contextual influences. The characterization of the THF loop–receptor interaction within the context of a self-assembling tectoRNA however allows the component parts to act in *trans*, thereby freeing it from the constraints present in the *cis* system.

The previously reported crystal structure 4LVV revealed two possible binding sites for THF derivatives—one of which was shown to bind at the interface of the loop–receptor interaction (27). Using assays where the riboswitch's ability to regulate transcription in the presence and absence of folinic acid, Trausch *et al.* demonstrated that the binding site associated with the loop–receptor interaction was more important than the one associated with the three-way junction (27). As earlier indicated, a second reported crystal structure (3SUX) representing a different sequence variation of the THF riboswitch did not elucidate a FA binding site at the level of the loop–receptor (26). This crystal structure however consisted of a dimer resulting from a strand invasion of the 5′ and 3′ ends of a pair of riboswitches. Given that the loop–receptor interaction takes place near the site of strand invasion in 3SUX, the lack of the second metabolite-binding site could have been the result of this crystal artifact. In this regard, the tectoRNA system provided an ideal system to assess the influence of FA on dimerization through the loop–receptor interaction alone.

Herein, the loop–receptor interaction associated with 4LVV was found to be responsive to FA while the interaction associated with 3SUX was not. These finding suggests that the THF interaction may be able to take alternate conformations—both having the ability to assemble but only one having the ability to recognize FA (Supplementary Figure S1B). While tectoRNA 3SUX was unresponsive, tectoRNA 980 (which is only one point mutation away from 3SUX) was found to be responsive to FA. Lead cleavage data associated with 980 also confirmed greater protection of the phosphodiester backbone region around the receptor in the presence of FA. This suggests that 980 likely adopt a conformation similar to the one observed in 4LVV when FA is present. With respect to 4LVV, the resulting tectoRNAs suggest that the THF loop–receptor is suboptimal when it comes to producing the most stable loop–receptor interaction in the absence of folate derivatives. Indeed, in absence of FA, the preferred length of stem connector for THF tectoRNAs is 11 bps rather than 9 bp as initially predicted by the 4LVV tectoRNA 3D model in presence of FA (Figure 4A and Supplementary Figure S2). In the presence of FA however, lead cleavage profiles show dramatic protection of the loop–receptor region in the 9 bp version (4LVV_9) over the 11-bp version (4LVV) (Figure 5). In the context of the biological function associated to the THF loop–receptor interaction, this is not altogether surprising. In the case of a dynamic system that relies on changing conformations, it would be more surprising if nature selected for the most stable or rigid structure. Nature time and again prefers structural modules that are metastable. For instance, the meta-stability of receptors and/or loops in the unbound state has been observed for the GAAA/11nt interaction (53), several other GNRA loop–receptors (37,38,40) and T-loop mediated interactions (1,54). Other recurrent RNA modules identified in the ribosome such as the A-minor modules (30) and right angle modules (2,32,55), have been shown to fold and assemble suboptimally outside the context of the ribosome. These examples suggest that metastability is at the root of metabolite responsive riboswitches (e.g. (23,25–27)). As shown herein, few sequence variations within the THF loop–receptor module offer access to metastable interactions within a range of 4–5 kcal/mol to modulate the regulatory function of the THF riboswitch (Figure 3).

Experimental characterization via tectoRNA assembly also provides a number of interesting insights with respect to RNA nanotechnology. This work represents only the second example of a self-assembling tectoRNA dimer system that takes advantage of a long-range interaction other than GNRA/receptor interactions (45). Out of a total of 51 THF loop/receptor tested, twenty of them have *K*_d_s below 100 nM and five of them have a *K*_d_ equal or below 26 nM (4LVV.4 ≤ 980.6 < 980.5 ≤ 980.10 < 980) in absence of folate at 2 mM magnesium. At 15 mM magnesium, the apparent *K*_d_s of these tectoRNAs are below the detection limit of 1 nM (data not shown). While our experiments indicate that folate derivatives are not required for tectoRNA assembly, they are shown to have the ability to increase dimerization by factors >70-fold in some cases. Interestingly, for 4LVV tectoRNA derivatives, the responsiveness of the THF tectoRNA is significantly enhanced in the 9-bp version (4LVV_9) over the 11-bp version (4LVV) by at least two orders of magnitude in presence of 0.5 mM Mg^2+^ at 20°C (Figure 5E(i)). This strongly suggests that tectoRNA responsiveness towards FA could be similarly increased for 980 derivatives. As such, the THF loop–receptor interface constitutes an interesting part for generating novel responsive nucleic acid nanostructures (e.g. (8,56–59)) for which FA responsiveness could be further modulated by varying temperature and salt concentration. Finally, modeling and characterization of tectoRNAs reveal that the long-range interaction found in the THF riboswitch could potentially work in combination with other known loop–receptor interactions such as the GNRA/11nt motif or other long-range interactions such as those associated with the ZTP riboswitch (60–63) or Guanidine-II riboswitch (64,65), which both hold potential as responsive modules for RNA assembly. As a matter of fact, we have obtained preliminary data indicating that an heterodimer combining the 980 THF loop/receptor interaction with the GAAA/11nt interaction (Supplementary Figure S1) can assemble with a *K*_d_ of 38 ± 7 nM without FA, and with a K_d_ below the detection limit of 1 nM in presence of 200 μM FA and 2 mM Mg(OAc)_2_ at 7°C (Supplementary Table S4). As the number and variety of long-range interactions continues to expand, the potential to mix and match modules to create new recombinant constructs with novel properties promises to find new and exciting applications in RNA nanotechnology and synthetic biology.

## Supplementary Material

gkz304_Supplemental_File
