# Supplementary material for: Responsive self-assembly of tectoRNAs with loop–receptor interactions from the tetrahydrofolate (THF) riboswitch
Source: Nucleic Acids Res. 2019 May 2;47(12):6439–51. doi: 10.1093/nar/gkz304 (PMC6614920; doi:10.1093/nar/gkz304)
Supplement: gkz304_Supplemental_File [file gkz304_supplemental_file.pdf]

SUPPLEMENTARY DATA for

Responsive self-assembly of tectoRNAs with loop-receptor interactions from the tetrahydrofolate (THF) riboswitch

Charles Mitchell[1], Julio Polanco[2], Laura DeWald[1], Dustin Kress[1], Luc Jaeger[2,#] and Wade W. Grabow[1,#]
[1]*Department of Chemistry and Biochemistry, Seattle Pacific University, Seattle, WA 918119-1997, USA;*
[2]*Department of Chemistry and Biochemistry, Biomolecular Science and Engineering Program, University of California, Santa Barbara, CA 93106-9510, USA.*
# - to whom correspondence should be addressed: Wade W. Grabow, email: grabow@spu.edu, phone: 206-281-2016. Luc Jaeger, email: jaeger@chem.ucsb.edu, phone: (805) 893-3628.

A. Supplemental Materials and Methods

DNA Amplification: All tectoRNAs were synthesized from DNA duplexes generated by PCR using the appropriate forward, reverse, and template strands (Integrated DNA Technology). Primers were designed to having annealing temperatures between 56 and 60°C. Individual dsDNA templates were synthesized using Taq DNA polymerase (Thermo Fisher Scientific) using the accompanied 10X buffer (200mM Tris-HCl pH 8.4, 500mM KCl); Magnesium Chloride (50mM)) according to manufacturer's recommendations. The annealing temperature for PCR was set to 56°C. Typically, 35 rounds of PCR were used to generate an appropriate amount of dsDNA. The resulting dsDNA was purified by GenCatch™ PCR cleanup kit (Epoch Life Science). The general signature for the three DNA strands used to generate the dsDNA used for transcription were as follows:

```
                      R5'                L                    R3'
tectoRNA 5'- GGGACAGXXXXGGUAAACAGGGXXXXXXXXXXXUCCUGUUUACCXXXXXXXXCUGUCC -3'
tectoRNA.tmp 5'- GGACAGXXXXXXXXGGTAAACAGGAXXXXXXXXXXXCCCTGTTTACCXXXXCTGTCCC -3'
tectoRNA.fwd 5'- TTCTAATACGACTCACTATAGGGACAGXXXXGGTAAACAGGG - 3'
tectoRNA.rev 5'- GGACAGXXXXXXXXGGTAAA - 3'
```

Transcription: TectoRNAs were transcribed from resulting dsDNA templates using *in vitro* run-off transcription by T7 RNA polymerase (Thermo Fisher Scientific) using the accompanied 5X Transcription Buffer (200 mM Tris-HCl (pH 7.9 at 25°C), 30 mM MgCl2, 50 mM DTT, 50 mM NaCl and 10 mM spermidine). Reaction mixtures consisted of a total volume of 200 µL with the following composition:

Transcription buffer (5X) 40 µL

NTPs (25 mM each)	20 µL
DTT (100 mM)	2.0 µL
Inorganic Pyrophosphatase (0.1 U/µl)	6.0 µL
RNasin (40u/µl)	1.5 µL
T7 RNA polymerase (150 Units)	1-7.5 µL
H2O (nanopure) (final volume of mix is 160 µL per reaction)	
PCR DNA template purified	~40 µL

Reaction mixtures were incubated at 37°C for 4 hours. After 4 hours, 2 µL of RQ1 DNase (10 U/µl) (New England Biolabs) was added and mixture was incubated for an additional 20 minutes to degrade the DNA templates.

RNA Purification and Isolation: To each reaction mixture 75 µl of urea blue (0.05% bromophenol, 0.05% xylene cyanol, 8M urea, 20 mM EDTA) was added and samples were heated at 90°C for 2 minutes before being loaded onto a denaturing polyacrylamide gel (8-10%, acrylamide bisacrylamide (19:1), 8 M urea). Samples were run for approximately 1.5 hours at 35W. Gel pieces containing RNA were visualized by UV shadowing technique, removed, and placed in elution buffer (200 mM NaCl, 10 mM Tris pH 7.5, 0.5 mM NaEDTA) and shook at 5°C overnight. The next morning the liquid was removed from the sample tubes, 900 µL of 100% ethanol was added to each tube, and samples were stored at -20°C for 2 hours to precipitate the RNA. Samples were spun for 1 hour at 12,000 rpm. The majority of the liquid was removed, washed with 90% ethanol, and spun at 12,000 rpm to stabilize the RNA pellet. This liquid was removed and sample was washed one final time. RNA was reconstituted in nanopure water and concentrations were determined using a nanodrop UV-Vis spectrometer (Thermo Fisher Scientific).

B. Supplemental Tables

Table S1: Alignment of unique loop-receptor sequences. Adapted from (1). The 5' receptor, loop, and 3' receptor regions are separated by dashes. Accession numbers are shown where the last bolded three number designations in blue represent the working name of each given sequence. Sequence signatures are grouped according to uniqueness by black dashed lines. For example, sequence 980 has the same loop-receptor sequence as the first 12 other THF riboswitches. For consensus sequence: red, 95% conserved; black, 85% conserved; gray, 75% conserved; R= A or G; Y= U or C; W=A or U. For alignment: underlined nts, loop-receptor duplex with WC and non-canonical bps; gray shading, WC bps; green shading, G-U bps.

```
2D                                          L1-2 P3      L3      P3′   L2-1
Consensus                                   AGUA-GRAY.GGGRWGUUGYY-GCAUYCCG
>NZ_AAYG02000031.1/153078-152980            AGUA-GGAC.GGGGAGUUGCC-GCAUCCCG
>NZ_AAXG02000014.1/43071-43187              AGUA-GGAC.GGGGAGUUGUC-GCAUCCCG
>BABE01004909.1/284-381                     AGUA-GAAC.GGGGAGUUGUC-GCAUCCCG
>NZ_ABAW02000025.1/72799-72896              AGUA-GAAC.GGGGAGUUGUC-GCAUCCCG
>NC_009706.1/3903929-3904044                AGUA-GAAC.GGGGAGUUGUC-GCAUCCCG
>BABA01009937.1/46-141                      AGUA-AGAC.GGGGAGUUGCU-GCAUCCCG
>BAAV01010818.1/618-715                     AGUA-GUAC.GGGGAGUUGAC-GCAUCCCG
>BAAX01022545.1/141-43                      AGUA-CAAC.GGGGAGUUGUG-GCAUCCCG
>NZ_ABED02000024.1/137574-137672            AGUA-CAAC.GGGGAGUUGUG-GCAUCCCG
>BABG01004557.1/3-100                       AGUA-CAAC.GGGGAGUUGUG-GCAUCCCG
>BAAX01002679.1/2103-2006                   AGUA-CAAC.GGGGAGUUGUG-GCAUCCCG
>BAAU01000940.1/627-724                     AGUA-CAAC.GGGGAGUUGUG-GCAUCCCG
>hgutS7_s7_164370/285-380                   AGUA-ACAC.GGGGAGUUGGU-GCAUCCCG
>NZ_AASF01001777.1/1578-1480                AGCA-GGAC.GGGGAGUUGUC-GCAUCCCG
>BABB01000157.1/63-158                      AGUA-GAAC.AGGGAGUUGUC-GCAUCCCG
>BABF01004573.1/52-147                      AGUA-GAAC.AGGGAGUUGUC-GCAUCCCG
>BABG01026644.1/586-688                     AGUA-GAAC.AGGGAGUUGUC-GCAUCCCG
>NZ_ABCC02000039.1/266095-266205            AGUA-GGAC.AGGGAGUUGUC-GCAUCCCG
>hgutS7_s7_168056/60-187                    AGUA-GGAC.AGGGAGUUGUC-GCAUCCCG
>BAAU01016335.1/416-525                     AGUA-GAAC.AGGGAGUAGUC-GCAUCCCG
>NZ_ABCA03000044.1/64560-64460 (3SUX)       AGUA-GGAC.GGGGAGUUGUC-GCAUCUCG
>NC_008525.1/1424554-1424642                AGUA-AGAC.GGAGAGUUGCU-GCAUCUCG
>NZ_ABDQ01000031.1/13491-13593              AGUA-GAAC.GGGAAGUUGUC-GCAUUCCG
>NC_008593.1/1647712-1647607                AGUA-GAAC.GGGAAGUUGUC-GCAUUCCG
>NZ_AAVC01000075.1/8139-8248                AGUA-GGAC.GGGAAGUUGCC-GCAUUCCG
>NC_009699.1/914572-914676                  AGUA-GGAC.GGGAAGUUGUC-GCAUUCCG
>NC_009495.1/892227-892331                  AGUA-GGAC.GGGAAGUUGUC-GCAUUCCG
>BAAZ01020636.1/794-894                     AGUA-AAACGGGGAAGUUGUU-GCAUUCCG
>BABE01001528.1/194-88                      AGUA-GGCU.GGGGAGUCGCC-GCA.CCCG
>hgutS8_s8_179091/613-719                   AGUA-GGCU.GGGGAGUCGCC-GCA.CCCG
>NZ_AAVO02000004.1/164832-164736            AAUA-GAAU.GGGGAGUUGUC-GCA.CCCG
>BABE01017430.1/618-522                     AAUA-GAAU.GGGGAGUUGUC-GCA.CCCG
>hgutS7_s7_164211_1/947-835                 AAUA-GGAU.GGGGAGUUGCC-GCA.CCCG
>BAAZ01000245.1/2377-2470                   AGUA-GGAU.GGGAGGUUGCC-GCAA.CCG
>NC_009633.1/4463946-4464038                AGUA-GGAU.GGGAAGUUGCU-GCGU.CCA
>NC_009922.1/2619446-2619544                AGUA-GGAU.GGGAAGUUGCC-GCAU.CCG
>NC_004557.1/1800312-1800211                AGUA-GGAU.AGGAUGUUGCC-GCUU.CCG
>NC_003030.1/2534634-2534731                AGUA-GGAU.GGGAAGUUGUC-GCUU.CCG
>BABD01033677.1/236-329                     AGUA-GGAU.GGGAAGUUGUC-GCUU.CCG
>NC_009706.1/1524321-1524441                AGUA-GAAU.GGGAGGUUGUC-GCUU.CCG
>NC_004350.1/559729-559641 (4LVV)           AGUA-GAAU.GGGAUGUCGUC-GCAU.CCG
>NZ_ABEE02000015.1/221133-221224            AGUA-AGAU.GGGAUGUUGCU-GCAU.CCA
>JCVI_SCAF_1101668087879/886-799            AGUA-AAAU.GGGUUGUUGUU-GCAA.CCG
>NC_009442.1/125748-125890                  AGUA-GGAU.GGGGUGUUGCC-GCAU.CCG
>NZ_AAOZ01000019.1/12278-12377              AGUA-GGAU.GGGACGUUGCC-GCGU.CCG
>NC_004668.1/903576-903739                  AGUA-GGAU.GGGAUGUUGCC-GCAUU.CG
>NZ_AAAK03000102.1/3116-3216                AGUA-GGAU.GGGAUGUUGCC-GCAUU.CG
>NC_009633.1/4839802-4839709                AGUA-GGAU.GGAACGUUGUA-GCGUU.CG
>NC_007576.1/348925-349019                  AGUA-GAAC.GGAAUGU.GAA-GCAUU.CG
>NC_008526.1/2247840-2247748                AGUA-GAAC.GGAAUGU.GAA-GCAUU.CG
>NC_008497.1/852209-852116                  AGUA-GAAC.GCAAUGU.GAA-GCAUU.GG
>NZ_AAPZ02000001.1/1062273-1062367          GGUA-GAAC.GGAAUGU.GAA-GCAUU.CA
>NC_007929.1/1666649-1666554                GGUA-GAAC.GGAAUGU.GAA-GCAUU.CA
>NC_010609.1/1983266-1983360                GGUA-GAAC.GGAAUGU.GAA-GCAUU.CA
>NC_008531.1/123971-124060                  GGUA-AGAC.GGAAUGU.GAG-GUGUU.AG
>BABA01028836.1/106-1                       AGUA-ACCG.GGGACGU.GAC-GUCGUCCG
>NZ_AASF01001777.1/1578-1480                CUCA-GAGC.GGAGCGCUGUC-GCCUAUUU
```

Table S2. Average K_d and standard deviation for each molecule at varying stem lengths tested in TB buffer (89mM Tris-borate, pH 8.2, 50mM KCl, and 2mM Mg^{2+}) at 7°C. Further details are listed in the Material and Methods.

	Equilibrium Dissociation Constant (Kd in nM)		
THF loop-receptor	9 base pair	10 base pair	11 base pair
4LVV	492 (±74)	252 (±94)	98 (±8)
3SUX	> 20000	> 20000	2534 (±396)
980	550 (±204)	30 (±16)	26 (±6)
593	> 20000	18228 (±1902)	5148 (±474)
158	2624 (±664)	2052 (±128)	1190 (±268)

Table S3: Average Kd, ΔG, and ΔΔG values for tectoRNAs in the absence and presence of folinic acid (FA). $\Delta\Delta G_{FA}$ values correspond to the difference in ΔG between control (no folinic acid) and the ΔG of each respective construct in the presence of 200 µM FA. All Kds were measured at 7°C and 1mM Mg^{2+} except the 3SUX variants, which did not dimerize in these conditions and were instead tested at 2 mM Mg^{2+} to achieve dimer formation. Data represent the average and standard deviation from a minimum of three independent experiments. 4LVV stands for 4LVV_11 (Table S4).

Molecule	Kd (no FA)	ΔG (no FA)	Kd (+FA)	ΔG (+FA)	$\Delta\Delta G_{FA}$
980	1944 (±108)	-7.32 (±0.03)	28 (±2)	-9.67 (±0.04)	-2.35 (±0.05)
148	540 (±20)	-8.03 (±0.02)	104 (±28)	-8.94 (±0.15)	-0.91 (±0.15)
4LVV	1072 (±20)	-7.65 (±0.01)	86 (±12)	-9.05 (±0.08)	-1.40 (±0.08)
4LVV_9	3694 (±432)	-6.96 (±0.07)	74 (±6)	-9.13 (±0.05)	-2.17 (±0.08)
4LVV_9.1	2338 (±114)	-7.21 (±0.03)	978 (±30)	-7.69 (±0.02)	-0.48 (±0.03)
3SUX*	2534 (±396)	-7.17 (±0.09)	3918 (±1132)	-6.92(±0.16)	0.25 (±0.18)
3SUX.1*	13640 (±2694)	-6.23 (±0.11)	9786 (±2136)	-6.41 (±0.12)	-0.18 (±0.16)

Table S4: List of tectoRNA constructs tested in this study with their corresponding Kds and free energies. Data represent the average and standard deviation from a minimum of three independent experiments performed in presence of 2 mM $Mg(OAc)_2$ at 7°C.

Name	TectoRNA Sequence	Interaction	Kd nM	ΔG(kcal/mol)
4LVV_9	5'-gGGACAG**AGUA**GGUAAAC--GG**AU-GGGAUGUCG**UC--GUUUACC**GCAUC-CG**CUGUCC	5'AUGGGAUGUCG<br>3'GCCUACG	492(±74)	-8.08(±0.08)
4LVV_10	5'-gGGACAG**AGUA**GGUAAACA-GG**AU-GGGAUGUCG**UC-UGUUUACC**GCAUC-CG**CUGUCC	5'AUGGGAUGUCG<br>3'GCCUACG	250(±94)	-8.46(±0.21)
4LVV_11 (641)	5'-gGGACAG**AGUA**GGUAAACAGGG**AU-GGGAUGUCG**UCCUGUUUACC**GCAUC-CG**CUGUCC	5'AUGGGAUGUCG<br>3'GCCUACG	98(±8)	-8.98(±0.05)
3SUX_9	5'-gGGACAG**AGUA**GGUAAAC--GG**AC-GGGGAGUUG**UC--GUUUACC**GCAUCUCG**CUGUCC	5'ACGGGGAGUUG<br>3' GCUCUACG	>20000	N.A.
3SUX_10	5'-gGGACAG**AGUA**GGUAAACA-GG**AC-GGGGAGUUG**UC-UGUUUACC**GCAUCUCG**CUGUCC	5'ACGGGGAGUUG<br>3' GCUCUACG	>20000	N.A.
3SUX_11 (460)	5'-gGGACAG**AGUA**GGUAAACAGGG**AC-GGGGAGUUG**UCCUGUUUACC**GCAUCUCG**CUGUCC	5'ACGGGGAGUUG<br>3' GCUCUACG	2534(±396)	-7.17(±0.09)
980_9	5'-gGGACAG**AGUA**GGUAAAC--GG**AC-GGGGAGUUG**UC--GUUUACC**GCAUCCCG**CUGUCC	5'ACGGGGAGUUG<br>3' GCCCUACG	550(±202)	-8.02(±0.20)
980_10	5'-gGGACAG**AGUA**GGUAAACA-GG**AC-GGGGAGUUG**UC-UGUUUACC**GCAUCCCG**CUGUCC	5'ACGGGGAGUUG<br>3' GCCCUACG	30(±16)	-9.64(±0.30)
980_11	5'-gGGACAG**AGUA**GGUAAACAGGG**AC-GGGGAGUUG**UCCUGUUUACC**GCAUCCCG**CUGUCC	5'ACGGGGAGUUG<br>3' GCCCUACG	26(±6)	-9.72(±0.13)
158_9	5'-gGGACAG**AGUA**GGUAAAC--GG**AC-AGGGAGUUG**UC--GUUUACC**GCAUCCCG**CUGUCC	5'ACAGGGAGUUG<br>3' GCCCUACG	2624(±664)	-7.15(±0.14)
158_10	5'-gGGACAG**AGUA**GGUAAACA-GG**AC-AGGGAGUUG**UC-UGUUUACC**GCAUCCCG**CUGUCC	5'ACAGGGAGUUG<br>3' GCCCUACG	2052(±128)	-7.29(±0.03)
158_11	5'-gGGACAG**AGUA**GGUAAACAGGG**AC-AGGGAGUUG**UCCUGUUUACC**GCAUCCCG**CUGUCC	5'ACAGGGAGUUG<br>3' GCCCUACG	1190(±268)	-7.59(±0.12)
593_9	5'-gGGACAG**AGUA**GGUAAAC--GG**AC-GGGAAGUUG**UC--GUUUACC**GCAUUCCG**CUGUCC	5'ACGGGAAGUUG<br>3' GCCUUACG	>20000	N.A.
593_10	5'-gGGACAG**AGUA**GGUAAACA-GG**AC-GGGAAGUUG**UC-UGUUUACC**GCAUUCCG**CUGUCC	5'ACGGGAAGUUG<br>3' GCCUUACG	18226(±1904)	-6.07(±0.06)
593_11	5'-gGGACAG**AGUA**GGUAAACAGGG**AC-GGGAAGUUG**UCCUGUUUACC**GCAUUCCG**CUGUCC	5'ACGGGAAGUUG<br>3' GCCUUACG	5148(±472)	-6.77(±0.05)
736	5'-gGGACAG**AAUA**GGUAAACAGGG**AU-GGGGAGUUG**UCCUGUUUACC**GCA-CCCG**CUGUCC	5'AUGGGGAGUUG<br>3' GCCCACG	34(±20)	-9.57(±0.33)

736.1	5′-gGGACAG**AGUA**GGUAAACAGGG**AU-GGGGAGUUG**UCCUGUUUACC**GCA-CCCG**CUGUCC	5′AUGGGGAGUUG<br>3′ GCCCACG	84(±26)	-9.06(±0.17)
488	5′-gGGACAG**AGUA**GGUAAACAGGG**CU-GGGGAGUCG**UCCUGUUUACC**GCA-CCCG**CUGUCC	5′CUGGGGAGUCG<br>3′ GCCCACG	180(±54)	-8.64(±0.17)
488.1	5′-gGGACAG**AGUA**GGUAAACAGGG**AU-GGGGAGUCG**UCCUGUUUACC**GCA-CCCG**CUGUCC	5′AUGGGGAGUCG<br>3′ GCCCACG	256(±18)	-8.44(±0.04)
731	5′-gGGACAG**AGUA**GGUAAACAGGG**AU-GGGAAGUUG**UCCUGUUUACC**GCUUC-CG**CUGUCC	5′AUGGGAAGUUG<br>3′ GCCUUCG	46(±16)	-9.40(±0.19)
739	5′-gGGACAG**AGUA**GGUAAACAGGG**AU-GGGAUGUUG**UCCUGUUUACC**GCAUU-CG**CUGUCC	5′AUGGGAUGUUG<br>3′ GCUUACG	11368(±1180)	-6.33(±0.06)
799	5′-gGGACAG**AGUA**GGUAAACAGGG**AU-GGGUUGUUG**UCCUGUUUACC**GCAAC-CG**CUGUCC	5′AUGGGUUGUUG<br>3′ GCCAACG	182(±30)	-8.63(±0.09)
038	5′-gGGACAG**AGUA**GGUAAACAGGG**AU-GGGAAGUUG**UCCUGUUUACC**GCGUC-CA**CUGUCC	5′AUGGGAAGUUG<br>3′ ACCUGCG	184(±16)	-8.63(±0.05)
038.1	5′-gGGACAG**AGUA**GGUAAACAGGG**AU-GGGAAGUUG**UCCUGUUUACC**GCGUC-CG**CUGUCC	5′AUGGGAAGUUG<br>3′ GCCUGCG	42(±20)	-9.45(±0.26)
224	5′-gGGACAG**AGUA**GGUAAACAGGG**AU-GGGAUGUUG**UCCUGUUUACC**GCAUC-CA**CUGUCC	5′AUGGGAUGUUG<br>3′ ACCUACG	50(±22)	-9.35(±0.24)
224.1	5′-gGGACAG**AGUA**GGUAAACAGGG**AU-GGGAUGUUG**UCCUGUUUACC**GCAUC-CG**CUGUCC	5′AUGGGAUGUUG<br>3′ GCCUACG	28(±4)	-9.68(±0.08)
709	5′-gGGACAG**AGUA**GGUAAACAGGG**AU-GGAACGUUG**UCCUGUUUACC**GCGUU-CG**CUGUCC	5′AUGGAACGUUG<br>3′ GCUUGCG	362(±54)	-8.25(±0.08)
544	5′-gGGACAG**AGUA**GGUAAACAGGG**AU-GGGAAGUUG**UCCUGUUUACC**GCAUC-CG**CUGUCC	5′AUGGGAAGUUG<br>3′ GCCUACG	180(±14)	-8.64(±0.04)
544.1	5′-gGGACAG**AGAU**GGUAAACAGGG**AU-GGGAAGUUG**UCCUGUUUACC**GCAUC-CG**CUGUCC	5′AUGGGAAGUUG<br>3′ GCCUACG	50(±20)	-9.35(±0.22)
211	5′-gGGACAG**AGUA**GGUAAACAGGG**AU-AGGAUGUUG**UCCUGUUUACC**GCUUC-CG**CUGUCC	5′AUAGGAUGUUG<br>3′ GCCUUCG	2218(±118)	-7.24(±0.03)
367	5′-gGGACAG**GGUA**GGUAAACAGGG**AC-GGAAUGU-G**UCCUGUUUACC**GCAUU-CA**CUGUCC	5′ACGGAAUGUG<br>3′ ACUUACG	>20000	N.A.
060	5′-gGGACAG**GGUA**GGUAAACAGGG**AC-GGAAUGU-G**UCCUGUUUACC**GUGUU-AG**CUGUCC	5′ACGGAAUGUG<br>3′ GAUUGUG	>20000	N.A.
019 (367.1)	5′-gGGACAG**AGUA**GGUAAACAGGG**AC-GGAAUGU-G**UCCUGUUUACC**GCAUU-CG**CUGUCC	5′ACGGAAUGUG<br>3′ GCUUACG	8580(±446)	-6.49(±0.03)
061	5′-gGGACAG**AGUA**GGUAAACAGGG**CG-GGGACGU-G**UCCUGUUUACC**GUCGUCCG**CUGUCC	5′CGGGGACGUG<br>3′ GCCUGCUG	80(±20)	-9.09(±0.14)

470	5′-gGGACAG**AGUA**GGUAAACAGGG**AU-GGGAGGUUG**UCCUGUUUACC**GCAAC-CG**CUGUCC	5′AUGGGAGGUUG<br>3′ GCCAACG	2700(±642)	-7.13(±0.13)
148	5′-gGGACAG**AGCA**GGUAAACAGGG**AC-GGGGAGUUG**UCCUGUUUACC**GCAUCCCG**CUGUCC	5′ACGGGGAGUUG<br>3′ GCCCUACG	344(±96)	-8.28(±0.15)
441	5′-gGGACAG**AGUA**GGUAAACAGGG**AU-GGGAGGUUG**UCCUGUUUACC**GCUUC-CG**CUGUCC	5′AUGGGAGGUUG<br>3′ GCCUUCG	30(±2)	-9.64(±0.04)
894	5′-gGGACAG**AGUA**GGUAAACAGGG**ACGGGGAAGUUG**UCCUGUUUACC**GCAUUCCG**CUGUCC	5′ACGGGGAAGUUG<br>3′ GCCUUACG	738(±44)	-7.86(±0.03)
480	5′-gGGACAG**CUCA**GGUAAACAGGG**GC-GGAGCGCUG**UCCUGUUUACC**GCCUAUUU**CUGUCC	5′GCGGAGCGCUG<br>3′ UUUAUCCG	>20000	N.A.
480.1	5′-gGGACAG**AGUA**GGUAAACAGGG**AC-GGAGCGCUG**UCCUGUUUACC**GCCUAUUG**CUGUCC	5′ACGGAGCGCUG<br>3′ GUUAUCCG	>20000	N.A.
890	5′-gGGACAG**AGUA**GGUAAACAGGG**AU-GGGGUGUUG**UCCUGUUUACC**GCAUC-CG**CUGUCC	5′AUGGGGUGUUG<br>3′ GCCUACG	166(±48)	-8.69(±0.16)
980.1	5′--GGACAG**AGUA**GGUAAACAGGG**AU-GGGGAGUUG**UCCUGUUUACC**GCAUCCCG**CUGUCC	5′AUGGGGAGUUG<br>3′ GCCCUACG	84(±18)	-9.06(±0.12)
980.2	5′--GGACAG**AGUA**GGUAAACAGGG**AC-GGGGAGUUG**UCCUGUUUACC**G-AUCCCG**CUGUCC	5′ACGGGGAGUUG<br>3′ GCCCUAG	282(±56)	-8.39(±0.11)
980.3	5′--GGACAG**AGUA**GGUAAACAGGG**AC-GGGGAGUUG**UCCUGUUUACC**G-CUCCCG**CUGUCC	5′ACGGGGAGUUG<br>3′ GCCCUCG	46(±6)	-9.40(±0.07)
980.4	5′--GGACAG**AGUA**GGUAAACAGGG**AC-GGGGAGUUG**UCCUGUUUACC**GCCUCCCG**CUGUCC	5′ACGGGGAGUUG<br>3′ GCCCUCCG	56(±12)	-9.29(±0.12)
980.5	5′--GGACAG**AGUA**GGUAAACAGGG**AU-GGGGAGUUG**UCCUGUUUACC**G-AUCCCG**CUGUCC	5′AUGGGGAGUUG<br>3′ GCCCUAG	22(±2)	-9.81(±0.05)
980.6	5′--GGACAG**AGUA**GGUAAACAGGG**AU-GGGGAGUUG**UCCUGUUUACC**GC-UCCCG**CUGUCC	5′AUGGGGAGUUG<br>3′ GCCCUCG	20(±2)	-9.86(±0.06)
980.7	5′-gGGACAG**AGUA**GGUAAACAGGG**AC-GAGGAGUUG**UCCUGUUUACC**GCAUCCCG**CUGUCC	5′ACGAGGAGUUG<br>3′ GCCCUACG	>20000	N.A.
980.8	5′-gGGACAG**AGUA**GGUAAACAGGG**AC-GGGGAGUUG**UCCUGUUUACC**GCAUCCUG**CUGUCC	5′ACGGGGAGUUG<br>3′ GUCCUACG	>20000	N.A.
980.9	5′-gGGACAG**AGUA**GGUAAACAGGG**AC-GAGGAGUUG**UCCUGUUUACC**GCAUCCUG**CUGUCC	5′ACGAGGAGUUG<br>3′ GUCCUACG	122(±24)	-8.86(±0.11)
980.10	5′-gGGACAG**AGUA**GGUAAACAGGG**AC-CGGGAGUUG**UCCUGUUUACC**GCAUCCCG**CUGUCC	5′ACCGGGAGUUG<br>3′ GCCCUACG	22(±2)	-9.81(±0.05)
980.11	5′-gGGACAG**AGUA**GGUAAACAGGG**AC-GCGGAGUUG**UCCUGUUUACC**GCAUCCGG**CUGUCC	5′ACGCGGAGUUG<br>3′ GGCCUACG	30(±16)	-9.64(±0.30)

980.12	5'-gGGACAGAGUAGGUAAACAGGGAC-GGCGAGUUGUCCUGUUUACCGCAUCGCGCUGUCC	5'ACGGCGAGUUG<br>3' GCGCUACG	790(±298)	-7.82(±0.21)
980.13	5'-gGGACAGAGCAGGUAAACAGGGAC-GGGGAGCUGUCCUGUUUACCGCAUCCCGCUGUCC	5'ACGGGGAGCUG<br>3' GCCCUACG	1086(±54)	-7.64(±0.03)
980.14	5'-gGGACAGAGUAGGUAAACAGGGAC-GGAGAGUUGUCCUGUUUACCG-AUCUCGCUGUCC	5'ACGGAGAGUUG<br>3' GCUCUAG	36(±18)	-9.54(±0.28)
593.1	5'-gGGACAGAGUAGGUAAACAGGGAC-GGGGAGUUGUCCUGUUUACCGCAUUCCGCUGUCC	5'ACGGGGAGUUG<br>3' GCCUUACG	364(±126)	-8.25(±0.19)
4LVV.1	5'-gGGACAGAGUAGGUAAACAGGGAU-GGGUUGUCGUCCUGUUUACCGCAAC-CGCUGUCC	5'AUGGGUUGUCG<br>3' GCCAACG	2194(±116)	-7.25(±0.03)
4LVV.2	5'-gGGACAGAGUAGGUAAACAGGGAU-GGGAAGUCGUCCUGUUUACCGCAUC-CGCUGUCC	5'AUGGGAAGUCG<br>3' GCCUACG	8822(±988)	-6.48(±0.06)
4LVV.3	5'-gGGACAGAGUAGGUAAACAGGGAU-GGGUAGUCGUCCUGUUUACCGCAAC-CGCUGUCC	5'AUGGGUAGUCG<br>3' GCCAACG	>20000	N.A.
4LVV.4	5'-gGGACAGAGUAGGUAAACAGGGAU-GGGAAGUCGUCCUGUUUACCGCUUC-CGCUGUCC	5'AUGGGAAGUCG<br>3' GCCUUCG	20(±2)	-9.86(±0.06)
4LVV_9.1	5'-gGGACAGAGCAGGUAAAC--GGAU-GGGAUGUCGUC--GUUUACCGCAUC-CGCUGUCC	5'AUGGGAUGUCG<br>3' GCCUACG	494(±62)	-8.08(±0.07)
3SUX.1	5'-gGGACAGAGCAGGUAAACAGGGAC-GGGGAGUUGUCCUGUUUACCGCAUCUCGCUGUCC	5'ACGGGGAGUUG<br>3' GCUCUACG	13640(±2634)	-6.23(±0.11)
377	5'-gGGACAGAGUAGGUAAACAGGGAU-GGGACGUUGUCCUGUUUACCGCGUC-CGCUGUCC	5'AUGGGACGUUG<br>3' GCCUGCG	Not tested	
525	5'-gGGACAGAGUAGGUAAACAGGGAC-AGGGAGUAGUCCUGUUUACCGCAUCCCGCUGUCC	5'AUGGGACGUUG<br>3' GCCCUACG	Not tested	
642	5'-gGGACAGAGUAGGUAAACAGGGAC-GGAGAGUUGUCCUGUUUACCGCAUCUCGCUGUCC	5'ACGGAGAGUUG<br>3' GCUCUACG	Not tested	
116	5'-gGGACAGAGUAGGUAAACAGGGAC-GCAAUGU-GUCCUGUUUACCGCAUU-GGCUGUCC	5'ACGCAAUGUG<br>3' GGUUACG	Not tested	
**Heterodimer R+L**				
R980	5'-gGGACAGAGUAGAAGUUCCGGGG—AA-------ACUUGGUUCUUCGCAUCCCGCUGUCC		N.A.	
L980	5'----GGGAUAUGGAAGUUCCAGGAC-GGGGAGUUGUCUGGUUCUUCCUAAGUCCCU		N.A.	
R980+L980	**- FA**		38(±7)	
R980+L980	**+ FA**		<1	

C. Supplemental Figures

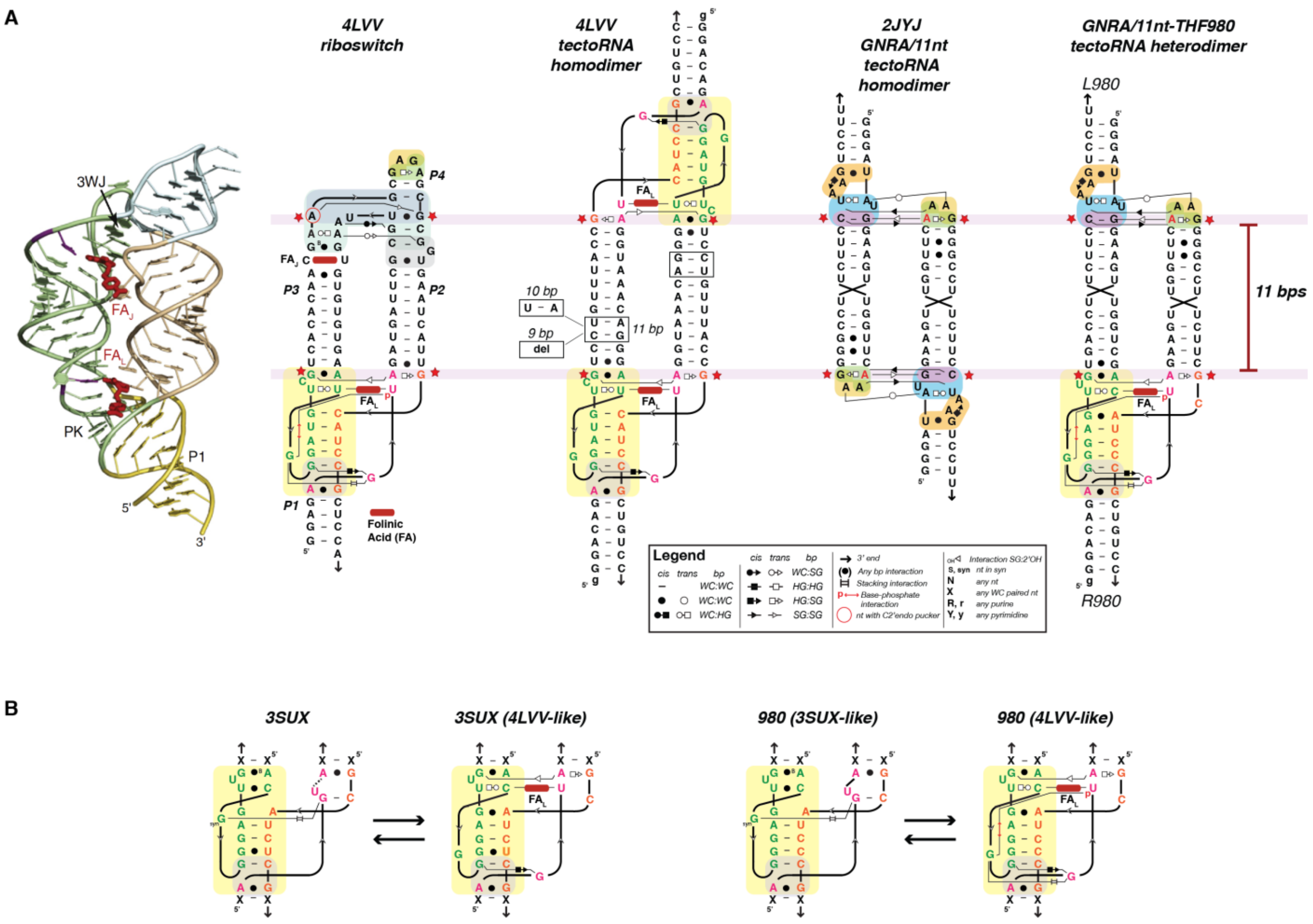

Figure S1: Comparison of the secondary structure diagrams of THF riboswitch [pdb_ID: 4LVV], THF tectoRNA homodimer, GAAA/11nt tectoRNA homodimer [according to pdb_ID: 2JYJ] and THF tectoRNA heterodimer. (**A**) The 3D schematic of the THF riboswitch on the far left is adapted from ref. (2). The distance separating the G:A mediated interactions (indicated by red stars) is 11bps in all structural contexts. The nomenclature for non-canonical base pairs and tertiary interactions are according to references (3,4). In the boxed legend: WC, Watson-Crick edge; HG, Hoogsteen edge; SG, shallow groove edge. (**B**) 2D diagrams of 3SUX and 980 interactions. The THF interaction might adopt two alternative structures. The conformations on the left and right correspond to the pattern of tertiary contacts observed in the crystallographic structures 3SUX and 4LVV, respectively. Note that 3SUX and 980 differ at only one nucleotide position.

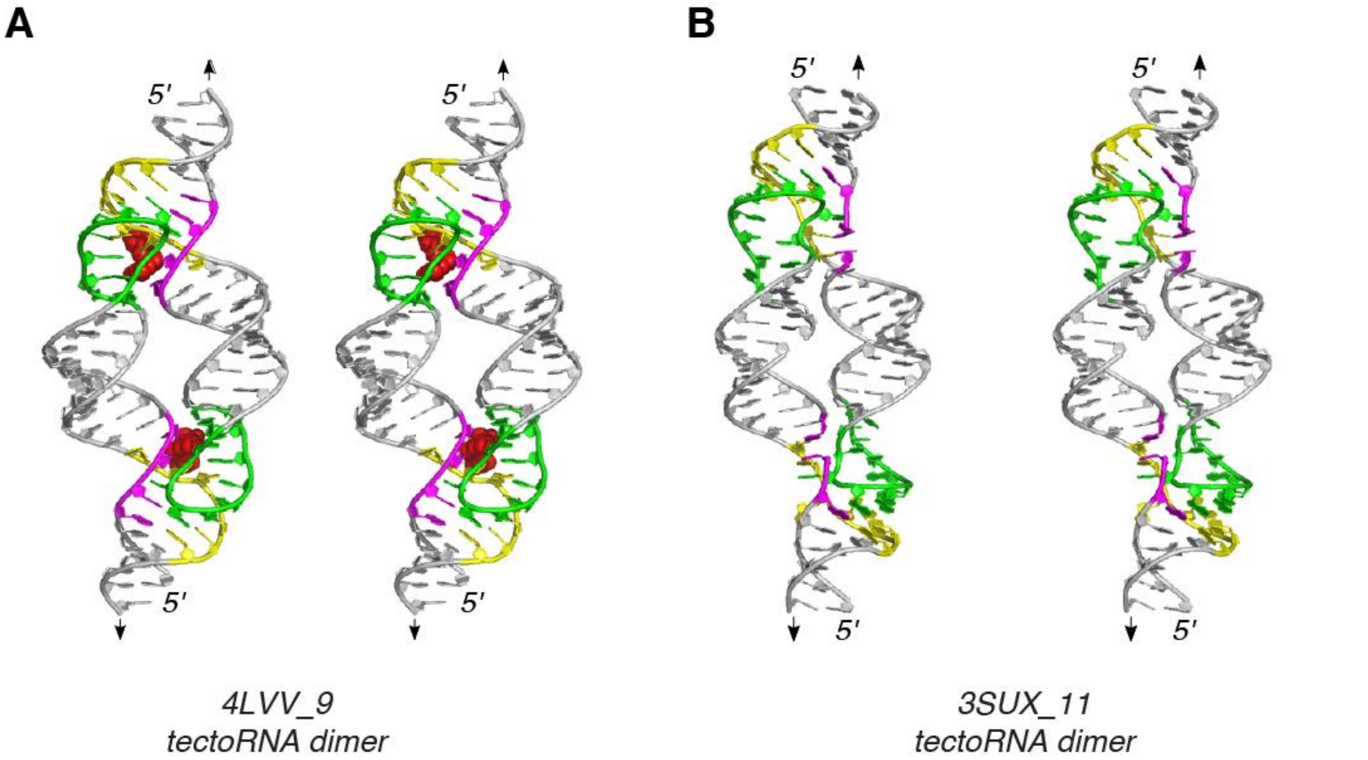

Figure S2: **Stereographic images of the 4LVV_9 and 3SUX_11 tectoRNA models.** (**A**) 4LVV tectoRNA dimer shows that a connector stem of 9 bps are preferable to the one of 11 bps for promoting assembly in presence of folinic acid (FA). (**B**) 3SUX tectoRNA dimer with connector stems of 11 bps, in absence of FA.

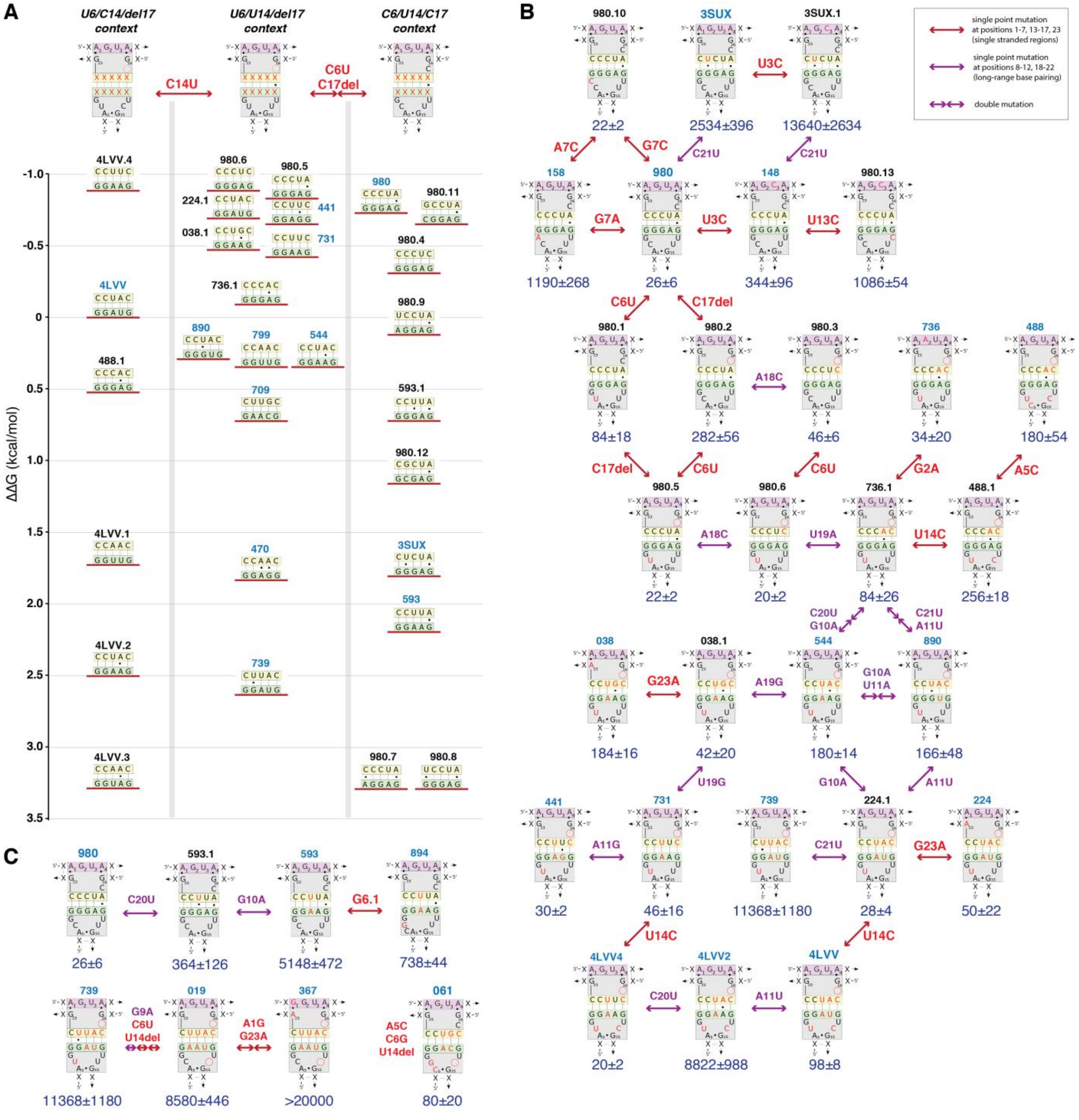

Figure S3: Self-assembly properties of THF loop-receptor interactions. **(A)** Comparison of the stability of long-range pairings in the 4LVV, 3SUX/980 and 441 contexts (4LVV context: U6, C14, del17; 3SUX/980 context: C6, U14, C17; 441 context: U6, U14, del17). Names in blue and black are for natural and artificial loop-receptors, respectively. **(B)** Mutational network of most loop-receptor variants: point mutations in single stranded regions 1-7, 13-17 and 23 are indicated in red, those in the long-range pairing (8-12; 18-22) are indicated in violet. The number of arrows between two 2D loop-receptor diagrams indicates the number of point mutations between them. Kd values (in nM) are indicated under each diagram in blue. **(C)** Additional loop-receptors in the U14del structural context (e.g. 019, 367 and 061) or with the mutation G6.1 (894).

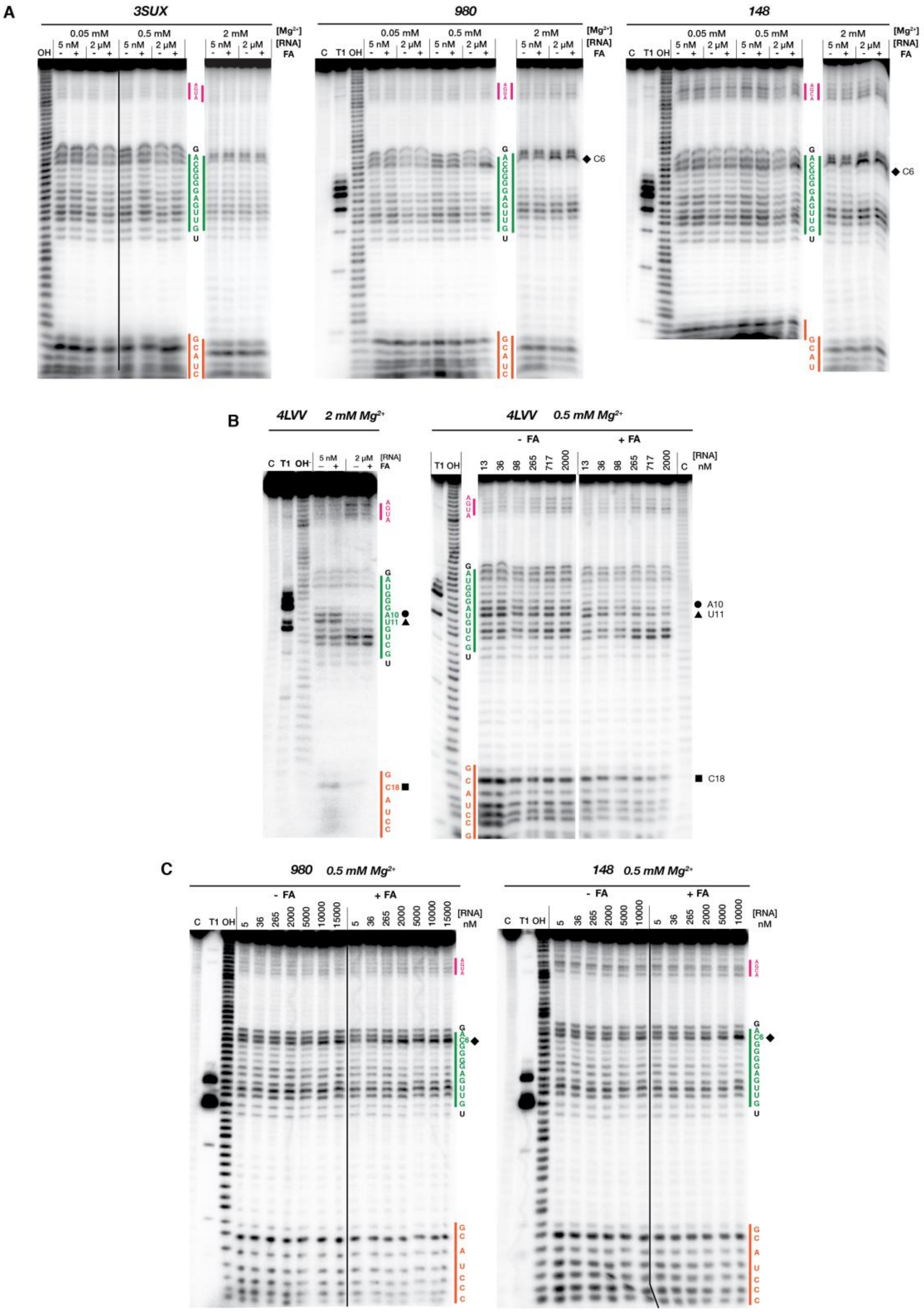

Figure S4: Lead(II)-induced cleavage analysis of 4LVV, 980 and 148 tectoRNAs in absence or presence of 200 µM FA. Typical gels are shown. Cleavage experiments were carried out with 10 mM Pb(II) for 5 min at various concentrations of Mg(OAc)$_2$ at 20°C as described in the Materials and Methods section. Lead cleavage patterns for: (**A**) 3SUX, 980 and 148 at 0.05, 0.5 and 2 mM Mg(OAc)$_2$, (**B**) 4LVV at 0.5 and 2 mM Mg(OAc)$_2$, (**C**) 980 and 148 at 0.5 mM Mg(OAc)$_2$.

D. Supplemental References

1. Ames, T.D., Rodionov, D.A., Weinberg, Z. and Breaker, R.R. (2010) A eubacterial riboswitch class that senses the coenzyme tetrahydrofolate. *Chemistry & biology*, **17**, 681-685.
2. Peselis, A. and Serganov, A. (2014) Themes and variations in riboswitch structure and function. *Biochimica et biophysica acta*, **1839**, 908-918.
3. Leontis, N.B. and Westhof, E. (2001) Geometric nomenclature and classification of RNA base pairs. *RNA*, **7**, 499-512.
4. Jaeger, L., Verzemnieks, E.J. and Geary, C. (2009) The UA_handle: a versatile submotif in stable RNA architectures. *Nucleic Acids Res.*, **37**, 215--230.
